# SGLT2 inhibitors mitigate kidney tubular metabolic and mTORC1 perturbations in youth-onset type 2 diabetes

**DOI:** 10.1172/JCI164486

**Published:** 2023-03-01

**Authors:** Jennifer A. Schaub, Fadhl M. AlAkwaa, Phillip J. McCown, Abhijit S. Naik, Viji Nair, Sean Eddy, Rajasree Menon, Edgar A. Otto, Dawit Demeke, John Hartman, Damian Fermin, Christopher L. O’Connor, Lalita Subramanian, Markus Bitzer, Roger Harned, Patricia Ladd, Laura Pyle, Subramaniam Pennathur, Ken Inoki, Jeffrey B. Hodgin, Frank C. Brosius, Robert G. Nelson, Matthias Kretzler, Petter Bjornstad

**Affiliations:** 1 Department of Internal Medicine, Division of Nephrology,; 2 Department of Computational Medicine and Bioinformatics, and; 3 Department of Pathology, University of Michigan, Ann Arbor, Michigan, USA.; 4 Department of Radiology,; 5 Department of Biostatistics and Informatics, and; 6 Department of Pediatrics, Section of Endocrinology, University of Colorado School of Medicine, Aurora, Colorado, USA.; 7 Department of Molecular and Integrative Physiology and; 8 Life Sciences Institute, University of Michigan, Ann Arbor, Michigan, USA.; 9 Division of Nephrology, The University of Arizona College of Medicine Tucson, Tucson, Arizona, USA.; 10 Chronic Kidney Disease Section, National Institute of Diabetes and Digestive and Kidney Diseases (NIDDK), Phoenix, Arizona, USA.; 11 Department of Medicine, Division of Renal Diseases and Hypertension, University of Colorado School of Medicine, Aurora, Colorado, USA.

**Keywords:** Metabolism, Nephrology, Chronic kidney disease, Diabetes, Transcription

## Abstract

The molecular mechanisms of sodium-glucose cotransporter-2 (SGLT2) inhibitors (SGLT2i) remain incompletely understood. Single-cell RNA sequencing and morphometric data were collected from research kidney biopsies donated by young persons with type 2 diabetes (T2D), aged 12 to 21 years, and healthy controls (HCs). Participants with T2D were obese and had higher estimated glomerular filtration rates and mesangial and glomerular volumes than HCs. Ten T2D participants had been prescribed SGLT2i (T2Di[+]) and 6 not (T2Di[–]). Transcriptional profiles showed SGLT2 expression exclusively in the proximal tubular (PT) cluster with highest expression in T2Di(–) patients. However, transcriptional alterations with SGLT2i treatment were seen across nephron segments, particularly in the distal nephron. SGLT2i treatment was associated with suppression of transcripts in the glycolysis, gluconeogenesis, and tricarboxylic acid cycle pathways in PT, but had the opposite effect in thick ascending limb. Transcripts in the energy-sensitive mTORC1-signaling pathway returned toward HC levels in all tubular segments in T2Di(+), consistent with a diabetes mouse model treated with SGLT2i. Decreased levels of phosphorylated S6 protein in proximal and distal tubules in T2Di(+) patients confirmed changes in mTORC1 pathway activity. We propose that SGLT2i treatment benefits the kidneys by mitigating diabetes-induced metabolic perturbations via suppression of mTORC1 signaling in kidney tubules.

## Introduction

The incedence of youth-onset type 2 diabetes (T2D) is rapidly rising among children and adolescents globally (1). It is associated with a more severe clinical course than youth-onset type 1 diabetes (T1D) and is characterized by the frequent appearance of diabetic kidney disease (DKD) in adolescence and young adulthood (2). The Treatment Options for Type 2 Diabetes in Adolescents and Youth follow-up study (TODAY2) documented a 15-year cumulative incidence of albuminuria of greater than 50% in young adults with T2D (3, 4). The young age and the relatively short follow-up period limited robust data on kidney failure in the TODAY2 cohort. However, among Southwest American Indians, in whom youth-onset T2D was first noted in the 1960s (5), kidney failure is nearly 5 times as high in midlife (ages 25–54) in those with youth-onset T2D than in those diagnosed with T2D later in life (6). Furthermore, adult Southwest American Indians with youth-onset T2D are more likely to have severe kidney structural lesions on kidney biopsy than those with adult-onset T2D of similar duration (7). These studies underscore the burden of DKD in youth-onset T2D and the lifetime risk of kidney failure and premature death in these patients.

Sodium-glucose cotransporter-2 (SGLT2) inhibitors (SGLT2i) are highly effective therapies that are revolutionizing the management of DKD in patients with T2D (8–11). Although not currently approved by the Food and Drug Administration for patients less than 18 years of age due to their high risk of developing DKD, SGLT2i are often prescribed off-label. However, the molecular mechanisms by which SGLT2i may confer kidney protection in youth-onset T2D are not well understood.

Accordingly, the objectives of this study were to characterize the effects of youth-onset T2D on kidney structure and function and understand the impact of SGLT2 inhibition on the morphological and molecular features of early kidney injury. To this end, research kidney biopsies donated by study participants were used for histological evaluation and single-cell RNA sequencing (scRNA-Seq) molecular profiling. Kidney transcriptomic perturbations corresponding to energy expenditure and substrate metabolism associated with youth-onset T2D were identified, and the cell type–specific impact of SGLT2i use on these transcripts was evaluated.

## Results

### Clinical and morphometric characteristics of cohort

**Figure 1:**
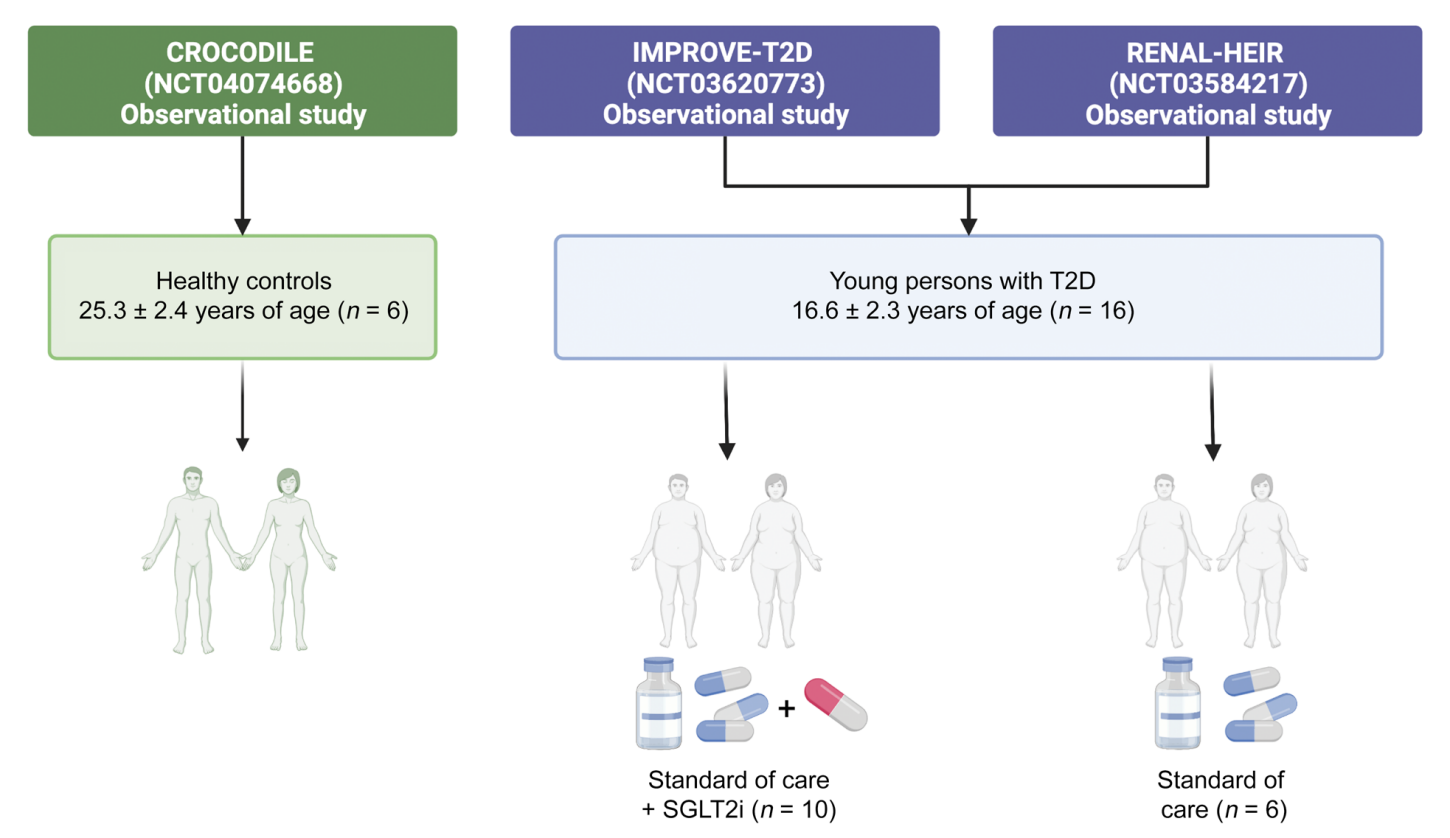
Participant data flow. For this study, young persons with type 2 diabetes treated with SGLT2 inhibitors or standard of care, from the IMPROVE-T2D and RENAL-HEIR observational studies, were included. Healthy controls from the CROCODILE study were included.

This study included data from 16 participants with T2D from the Impact of Metabolic Surgery on Pancreatic, Renal and Cardiovascular Health in Youth with Type 2 Diabetes (IMPROVE-T2D, NCT03620773) study and the Renal Hemodynamics, Energetics and Insulin Resistance in Youth Onset Type 2 Diabetes Study (Renal HEIR, NCT03584217) and 6 healthy controls (HCs) from the Control of Renal Oxygen Consumption, Mitochondrial Dysfunction, and Insulin Resistance (CROCODILE) study (NCT04074668) (Figure 1 and Supplemental Table 1; supplemental material available online with this article; https://doi.org/10.1172/JCI164486DS1). Participants with T2D were stratified by SGLT2i use: 10 had been treated with SGLT2i (T2Di[+]), and 6 had not (T2Di[–]). The median duration of SGLT2i treatment prior to kidney biopsy in the T2Di(+) group was 5 months. On average, participants with T2D (both T2Di[–] and T2Di[+]), were younger and had higher BMI and body fat, worse dyslipidemia, and higher estimated glomerular filtration rates (eGFR) than HCs. The participants with T2D were evenly distributed between males and females and had an average age of 17 ± 2 years. The T2Di(–) and T2Di(+) groups were similar in terms of clinical, laboratory, and morphometric features (Table 1). The average A1c in T2Di(–) patients was 6.9% ± 2.5%, while in T2Di(+) patients, it was 7.2% ± 1.4%. Moreover, 20% of T2Di(–) and 22% of T2Di(+) patients had albuminuria at the time of screening. Two T2Di(+) participants were prescribed empagliflozin; all others were prescribed canagliflozin.

Early kidney structural injury was observed in T2D patients, with greater mesangial matrix, mesangial nuclear count, mesangial volume, and glomerular volume than HCs (Supplemental Figure 1 and Table 2). However, fractional interstitial area was similar between T2D patients and HCs. Notably, mean glomerular volume in the T2Di(+) group was lower than in the T2Di(–) group, consistent with previously reported effects of SGLT2i on glomerular volume (12).

### scRNA-Seq results

**Figure 2:**
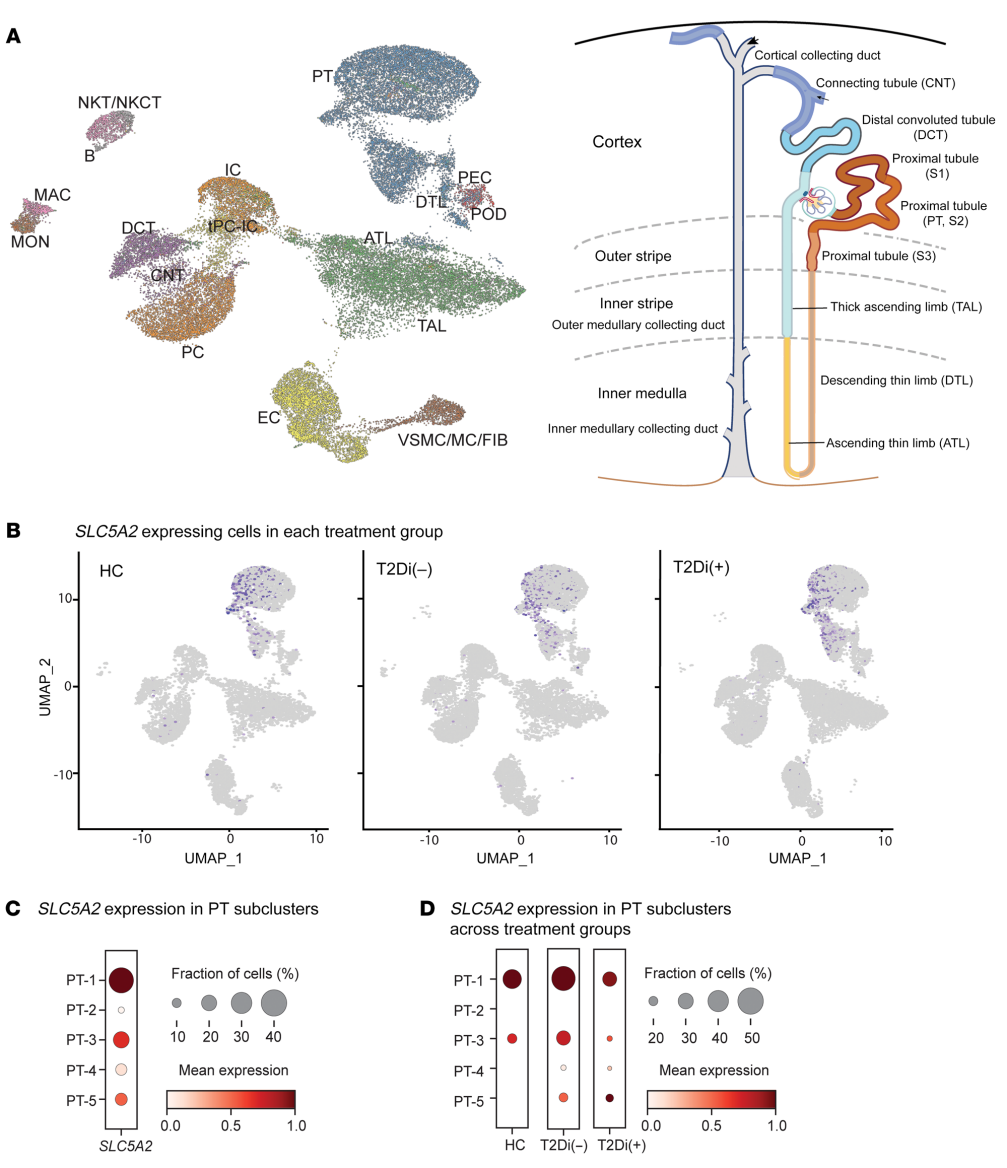
*SLC5A2* gene expression is limited to PT clusters. (**A**) UMAP projection of annotated cellular clusters from 3 groups, HCs (*n* = 6), T2Di (–) (*n* = 6), and T2Di(+) (*n* = 10), correspond to all major cell types in the nephron. Cluster identity and color are mapped onto the nephron schematic. (**B**) For all groups (HC, T2Di[–], and T2Di[+]), *SLC5A2* mRNA–expressing cells (purple dots) were limited to the PT cluster. (**C**) Size of dots, reflecting fraction of cells (%) expressing *SLC5A2* mRNA, and color intensity, indicating mean expression levels, varied across PT subclusters, with PT-1 having the highest expression and PT-2 and PT-4 having the lowest expression. (**D**) *SLC5A2* mRNA expression varied across the 3 groups, with T2Di(+) lower than T2Di(–) across all PT subclusters. ATL, ascending thin limb; DCT, distal convoluted tubule; CNT, connecting tubule; tPC-IC, transitioning intercalated/PCs; EC, endothelial cells; vSMC/MC/Fib, vascular smooth muscle cells/mesangial cells/fibroblasts; PEC, parietal epithelial cells; POD, podocytes; MAC, macrophages; MON, monocytes; B, B cells; NKT/NKCT, natural killer T cells/natural killer cells with T cells; PT, proximal tubule; DTL, descending thin limb; TAL, thick ascending limb; IC, intercalated cells; PC, principal cells.

scRNA-Seq profiles were generated from kidney biopsies from each of the participants (*n* = 22); 40,535 cells passed quality control requirements and were annotated to 18 clusters, representing all major cell types in the nephron (Figure 2A and Supplemental Figure 2A). Each cell cluster had a robust representation of the 3 biopsy groups (Supplemental Figure 2B).

The proximal tubular (PT) cell clusters also included robust representation from all PT segments. PT-1 mapped predominantly to S1 and S2 nephron segments using the Kidney Precision Medicine Project (KPMP) segment-specific marker set (13). Most of the other PT subclusters also mapped to the S1 and S2 nephron segments, with PT-4 contributing to the S1, S2, and S3 segments (Supplemental Figure 3, A and B). PT-5 had overall fewer cells and mostly mapped to S1 and S2 nephron segments (Supplemental Figure 3C). PT-4 had the highest expression of adaptive/maladaptive cell markers (Supplemental Figure 3D), while cycling cell markers had low expression across the subclusters (Supplemental Figure 3E) and degenerative markers were expressed relatively uniformly across the PT subclusters (Supplemental Figure 3F).

The mRNA expression of SGLT2 (*SLC5A2*) was enriched in PT (Figure 2B), and there were 5 PT subclusters (PT-1 to PT-5). The mRNA encoding the SGLT2i target protein, *SLC5A2*, was highest in PT-1, followed by PT-3 and PT-5 subclusters (Figure 2C). The proportion of cells in PT-1 expressing *SLC5A2* was higher in T2Di(–) patients compared with HCs and reverted to HC levels in T2Di(+) patients (Figure 2D).

### SGLT2 inhibitors associated with transcriptional changes throughout the kidney tubules

**Figure 3:**
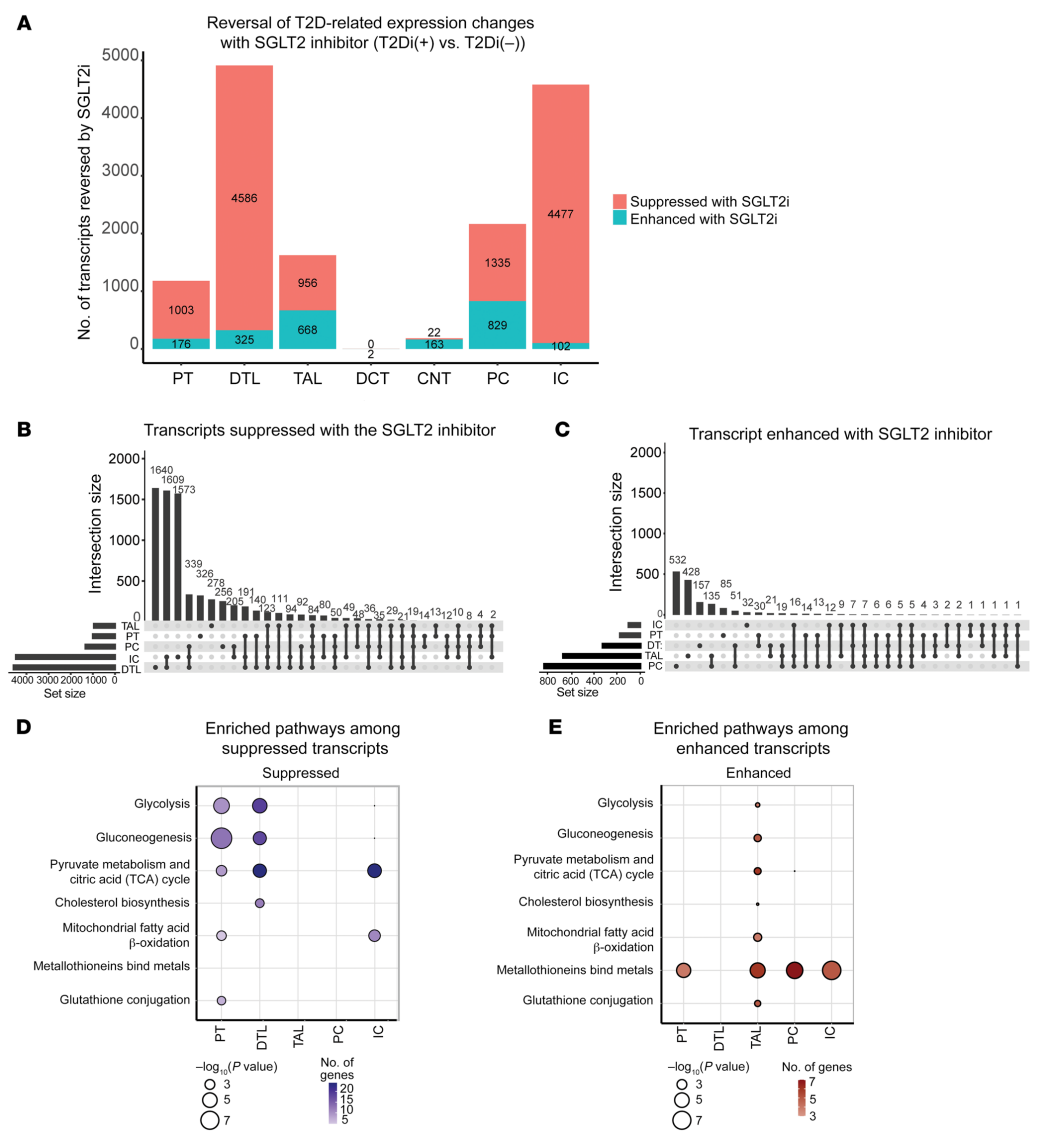
SGLT2 inhibition altered transcript expression in the majority of tubular cell segments. (**A**) Plot of the number of transcripts reversed, suppressed, or enhanced with SGLT2i shows that the majority of transcripts altered with SGLT2i were in distal nephron segments. The fold changes (log_2_FC) were calculated between 2 comparisons: T2Di(–) versus HCs and T2Di(+) versus T2Di(–). Transcripts were required to pass FDR-adjusted *P* values of less than 0.05 in T2Di(–) versus HCs and in T2Di(+) versus T2Di(–) to be considered reversed. (**B**) Upset plots indicate most transcripts suppressed with SGLT2i were in DTL. DTL and IC shared the greatest number of transcripts. (**C**) Most unique transcripts enhanced with SGLT2i were in PC, TAL, and DTL. PC and TAL had the greatest number of overlapping transcripts (*n* = 135). Using the Reactome database and Fisher’s exact test, (**D**) central metabolic pathways in PT, DTL, and IC were suppressed and (**E**) all central metabolic processes were enhanced in TAL. Metallothioneins were enhanced across all segments, except DTL.

Transcripts with statistically significant fold changes between T2Di(–) patients and HCs that also had significant fold changes in the opposite direction between T2Di(+) and T2Di(–) patients were considered “reversed” by treatment with SGLT2i (Supplemental Figure 4A). Transcripts with statistically significant fold changes between T2Di(–) patients and HCs were predominantly increased in the diabetic state (Supplemental Figure 4B). Despite *SLC5A2* being expressed only in the PT clusters, the largest number of transcripts reversed with SGLT2i treatment were in the descending thin limb (DTL), followed by intercalated cells (ICs), principal cells (PCs), thick ascending limb (TAL), and PT in descending order (Figure 3A). Very few podocytes and juxtaglomerular cells were recovered in the scRNA-Seq data, limiting investigations into tubuloglomerular feedback.

Upset plots (Figure 3, B and C) show the number of unique tubular segment–specific transcripts that were reversed and the changes shared between contiguous and noncontiguous tubular segments. The complete list of reversed transcripts is shown in Supplemental Table 2. Transcripts with reduced expression in T2Di(–) patients compared with HCs and increased expression in T2Di(+) compared with T2Di(–) patients were defined as “enhanced” transcripts (i.e., enhanced expression with SGLT2i). Transcripts that had increased expression in T2Di(–) patients compared with HCs and reduced expression in T2Di(+) compared with T2Di(–) patients were defined as “suppressed” transcripts (i.e., suppressed expression with SGLT2i). The number of suppressed transcripts was several-fold higher than the number of enhanced transcripts, most likely because T2Di(–) associated with several-fold more transcripts with increased expression compared with decreased expression. DTL, IC, and PC had the greatest number of unique and shared suppressed transcripts, while PC and TAL had the greatest number of shared enhanced transcripts. Figure 3, D and E, demonstrates pathways enriched within each tubular segment cell type with SGLT2i treatment. Transcripts within key metabolic pathways, such as glycolysis, gluconeogenesis, pyruvate metabolism, and citric acid cycle (TCA), were suppressed from PT to DTL segments. Transcripts within TCA cycle and β-oxidation of fatty acid pathways were suppressed in ICs, while PCs did not show suppression of transcripts associated with any metabolic process (Figure 3D). However, transcripts involved in these major metabolic pathways were enhanced in TAL with SGLT2i treatment (Figure 3E). Moreover, transcripts in metallothionein pathways, critical to mitigating damage from oxidative stress (14), were enhanced by SGLT2i across all tubular segments except DTL. The complete enrichment analysis results are available in Supplemental Table 3.

#### Proximal tubular cells.

**Figure 4:**
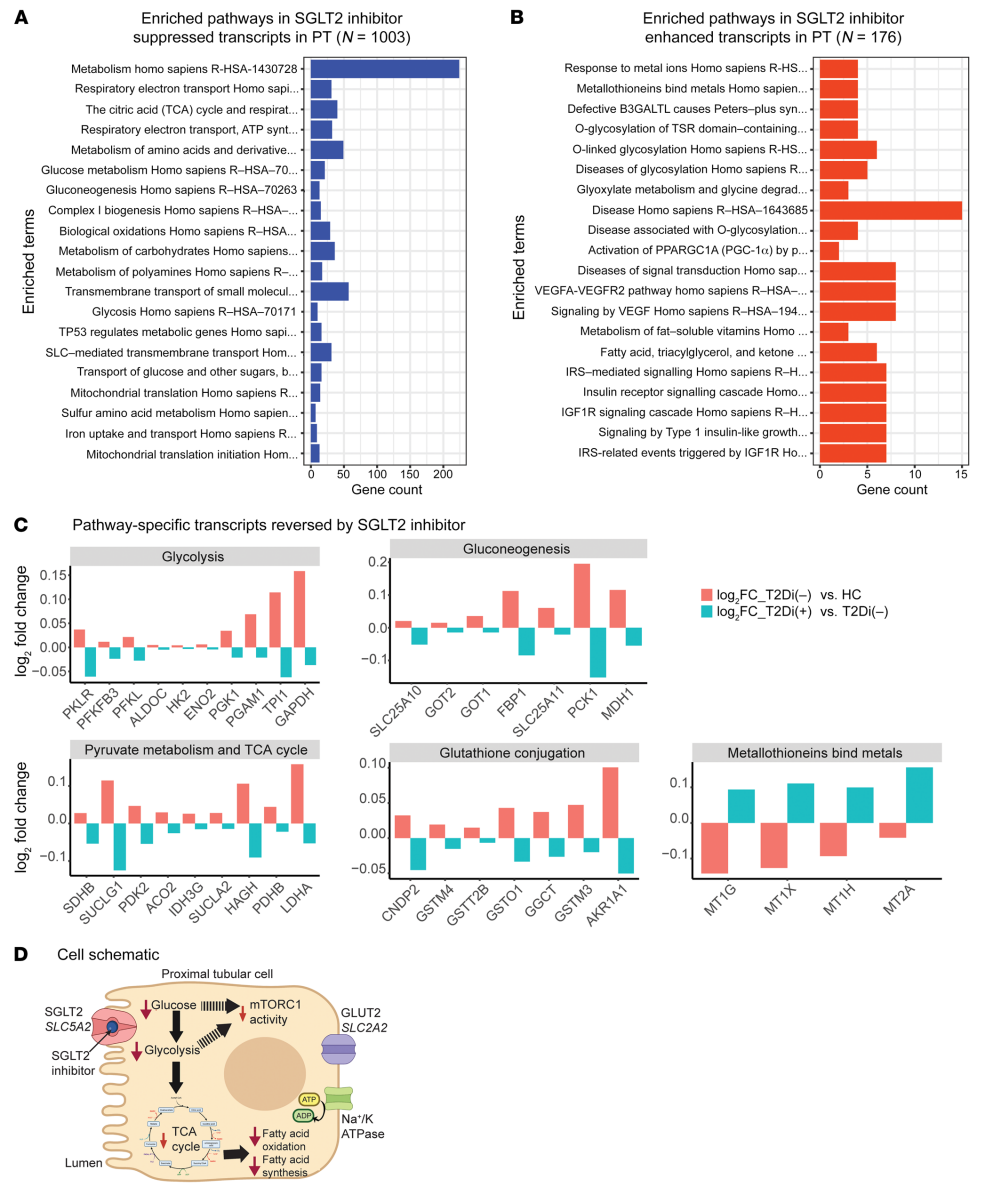
Suppression of central metabolic pathways with SGLT2 inhibition in PT. (**A**) Enrichment analysis using Reactome data of suppressed transcripts with SGLT2 inhibition showing metabolism has greatest number of altered transcripts (*n* > 200). (**B**) Pathway enrichment analysis using Reactome database of enhanced transcripts with SGLT2 inhibition. A pathway was considered significant if its *P* value was less than 0.05 and included at least 5 transcripts reversed by SGLT2i. (**C**) Bar plots showing transcript-level alterations (log_2_FC) when comparing T2Di(–) to HCs (pink) and T2Di(+) to T2Di(–) (blue). (**D**) Schematic summarizing the transcriptional changes in PT cells. SGLT2i impairs uptake of sodium and glucose into PT cells via *SLC5A2*, leading to decreased expression of glycolytic and TCA cycle transcripts. Decreased glucose uptake and glycolysis may decrease mTORC1 activity.

An examination of the top 20 significantly suppressed (Figure 4A) and enhanced (Figure 4B) pathways confirmed the suppression of central metabolism-related pathways in PT (Figure 4A) with SGLT2i treatment. The transcripts significantly reversed in each of the central metabolic pathways are presented in Figure 4C. Within glycolysis-specific enzymes, there was increased expression of transcripts encoding key rate-limiting enzymes in T2Di(–) patients compared with HCs, including hexokinase-2 (*HK2*), phosphofructokinase-liver (*PFKL*), and pyruvate kinase (*PKLR*). These transcripts were suppressed in the T2Di(+) group relative to T2Di(–) patients. Similarly, transcripts for other key glycolytic enzymes, such as aldolase (*ALDOC*), enolase (*ENO*), and *GAPDH*, were also suppressed with SGLT2i treatment. Furthermore, transcripts of specific rate-limiting enzymes of the gluconeogenic pathways were suppressed, including phosphoenolpyruvate carboxykinase (*PCK1*) and fructose 1,6-bisphosphatase (*FBP1*). These data suggest that SGLT2i treatment suppresses the elevated glycolysis and gluconeogenesis transcriptional profiles of PT cells induced by diabetes toward the HC state.

Downstream of glycolysis, SGLT2i treatment associated with the suppression of transcripts of rate-limiting enzymes that allow pyruvate to enter the TCA cycle, such as pyruvate dehydrogenase complex (*PDHB*) and pyruvate dehydrogenase kinase (*PDK2*) (15). Moreover, the aconitase (*ACO2*), isocitrate dehydrogenase (*IDH3G*), and components of succinyl Co-A synthase (*SUCLG1*, *SUCLA2*) transcripts were also suppressed. Transcripts critical to β-oxidation (16), such as acyl-CoA dehydrogenase long-chain (*ACADL*), showed diminished expression in T2Di(–) patients (versus HCs), but were relatively enhanced in T2Di(+) patients. Additionally, metallothionein transcripts, involved in mitigating oxidative stresses (14), were consistently enhanced by SGLT2i in PT (Figure 4C).

Differentially expressed gene (DEG) profiles in PT were visualized using protein-protein interaction networks to demonstrate the effects of SGLT2i on central metabolism and oxidative stress. Although SGLT2i reversed the direction of expression for many of these genes, they did not all completely revert to the HC state, as evidenced by the presence of significant fold changes when comparing T2Di(+) patients to HCs (Supplemental Figure 5).

To identify the differential impact of SGLT2i on PT cells, with and without *SLC5A2* expression, transcripts coexpressed with *SLC5A2* were investigated in HCs and T2Di(–) and T2Di(+) patients. PT cells with detectable *SLC5A2* expression (PT^+^) had more pathways suppressed with SGLT2i than PT cells where *SLC5A2* was not detected (PT^–^). While metabolism was suppressed in all PT cells, only PT^+^ cells showed enrichment for the TCA cycle. In PT^+^ cells, there were an insufficient number of enhanced transcripts for enrichment analysis, while in PT^–^ cells, VEGF and PI3K signaling were enriched (Supplemental Figure 6). Taken together, these data suggest that SGLT2 inhibition might be associated with a decreased rate of transepithelial glucose uptake leading to several metabolic changes, reflected in the reduction in glycolytic and TCA cycle transcripts, which could potentially lead to downstream suppression of mTORC1 pathway activity (Figure 4D).

#### Thick ascending limb cells.

**Figure 5:**
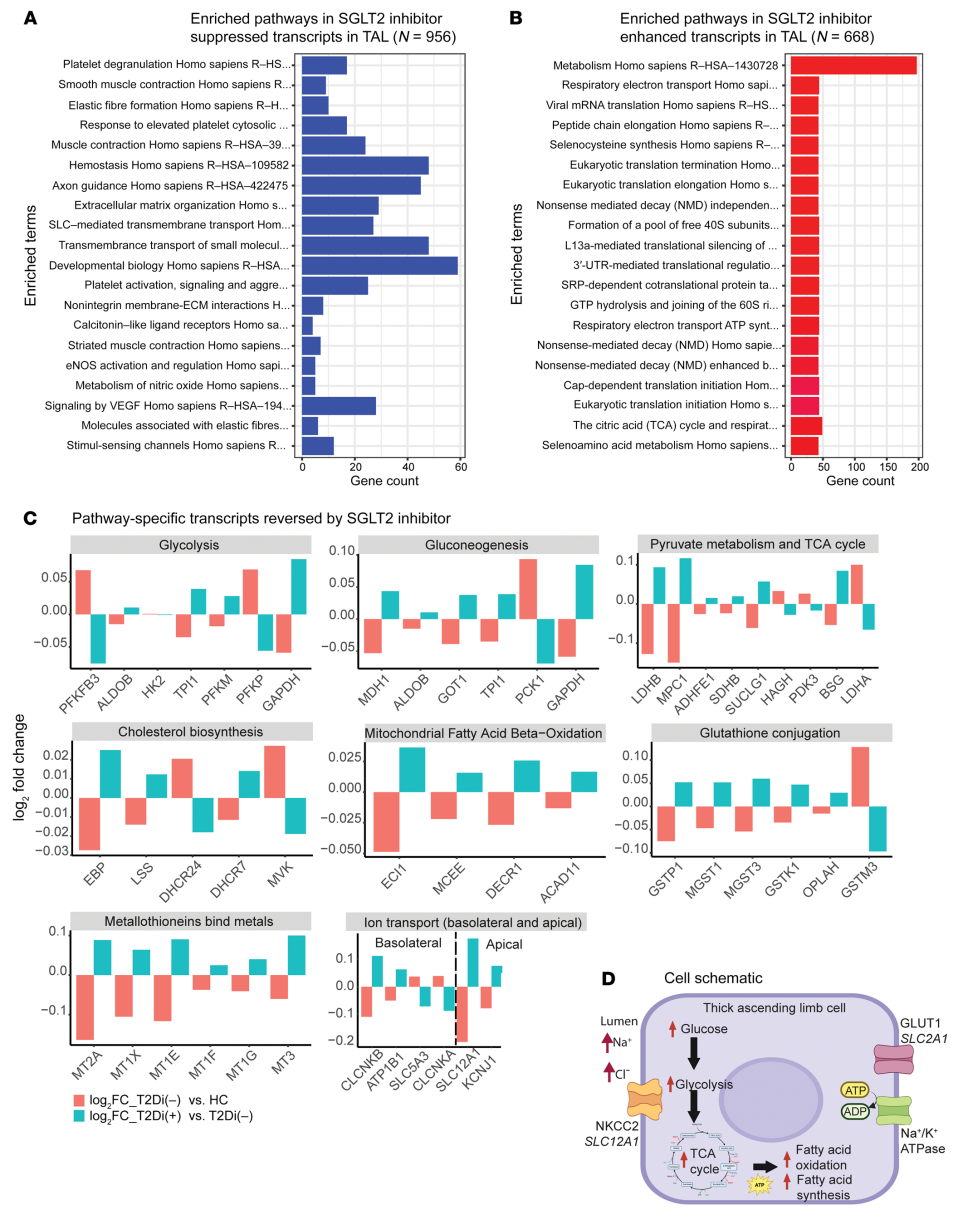
Enhancement of central metabolic pathways with SGLT2 inhibition in TAL. (**A**) Enrichment analysis using Reactome database of suppressed transcripts with SGLT2 inhibition. (**B**) Enrichment analysis using Reactome data of enhanced transcripts with SGLT2 inhibition showing metabolism has greatest number of altered transcripts (*n* > 200). A pathway was considered significant if its *P* value was less than 0.05 and included at least 5 transcripts reversed by SGLT2i. (**C**) Bar plots showing transcript-level alterations (log_2_FC) when comparing T2Di(–) to HCs (pink) and T2Di(+) to T2Di(–) (blue) (**D**) Schematic summarizing the transcriptional changes in TAL cells. Increased sodium-chloride delivery to tubular lumen would increase ATP consumption by sodium/potassium ATPase on the basolateral membrane. Increased energy needs could be met by increased expression of glycolytic, TCA cycle, and fatty acid oxidation transcripts.

The TAL response differed from that of other nephron segments, with central carbon metabolism being the top pathway significantly enhanced with SGLT2i, whereas in PT, central carbon metabolism was one of the top suppressed pathways (Figure 5, A and B). There were differences between the expression of key pathway transcripts for glycolysis in TAL compared with PT. For example, in PT, transcripts in the glycolysis pathway (*ALDOB*, *TPI1*, *PFKM*, *GAPDH*) were suppressed with SGLT2i treatment (Figure 4C), whereas in TAL, these transcripts or corresponding isozymes (*ALDOC*, *TPI1*, *PFKL*, *GAPDH*) were enhanced with SGLT2i treatment. Transcripts in the gluconeogenesis pathway were elevated in TAL with SGLT2i (Figure 3E). However, the transcript for the key gluconeogenic enzyme *PCK1* was suppressed with SGLT2i treatment (Figure 5C).

Similarly, TCA cycle transcripts in TAL and PT were affected in opposite directions with SGLT2i. In TAL, transcription of the lactate dehydrogenase B, mitochondrial pyruvate carrier 1, and components of succinate synthase and the succinate dehydrogenase complex (*LDHB*, *MPC1*, *SUCLG1*, and *SDHB*, respectively) were enhanced with SGLT2i. In contrast, these TCA components were suppressed with SGLT2i in PT. Moreover, uniquely to TAL, reversal of expression of the lanosterol synthase (*LSS*) and delta-7 sterol reductase (*DHCR7*) transcripts occurred with SGLT2i. These 2 transcripts are for rate-limiting enzymes in the de novo cholesterol biosynthesis pathway that convert oxidosqualene into lanosterol (*LSS*) and also convert 7-dehydrocholesterol into cholesterol (*DHCR7*). Several transcripts that oxidize branched and unsaturated fatty acids, including enoyl-CoA delta isomerase 1 (*ECI1*), methylmalonyl-CoA epimerase (*MCEE*), and acyl-CoA dehydrogenase family member 11 (*ACAD11*), had enhanced expression with SGLT2i treatment.

Different metallothionein members had enhanced expression with SGLT2i in PT and TAL. Transcripts in the glutathione conjugation pathway were suppressed in PT, but enhanced in TAL with SGLT2i. The direction of expression changes of glutathione conjugation pathway members in PT and TAL was also opposite (Figure 4C and Figure 5C), except for glutathione S-transferase mu 3 (*GSTM3*), which was suppressed in both segments with SGLT2i.

The expression of several transcripts related to basolateral and apical ion transport was reversed in TAL with SGLT2i treatment (Figure 5C). Of these, the electroneutral sodium/potassium/chloride transporter (NKCC2, *SLC12A1*), the apical tubular flow-mediated potassium rectifier channel (ROMK channel, *KCNJ1*), chloride voltage-gated channel B (*CLCNKB*), and a subunit of the basolateral sodium/potassium ATPase (*ATP1B1*) transcripts were enhanced by SGLT2i. Transcript expression of other ion transporters, such as *SLC5A3*, which encodes for the sodium-*myo*-inositol cotransporter, were suppressed with SGLT2i treatment. These data suggest that, with SGLT2 inhibition, TAL may need to increase sodium and chloride transport from the tubular lumen via NKCC2, resulting in transcriptional changes to support this process, including the concomitant increase in energy requirements by the sodium/potassium ATPase on the basolateral membrane (Figure 5D).

Similar enrichment analyses are summarized for DTL, PCs, and ICs in Supplemental Figure 7, with the complete enrichment analysis list for PT, TAL, DTL, PC, and IC nephron segments provided in Supplemental Table 3. Pathways that did not meet statistical significance for reversal with SGLT2 inhibition are a potential target for novel DKD therapies and are included in Supplemental Table 4.

### Validation of findings in T2D murine models treated with SGLT2 inhibitors

**Figure 6:**
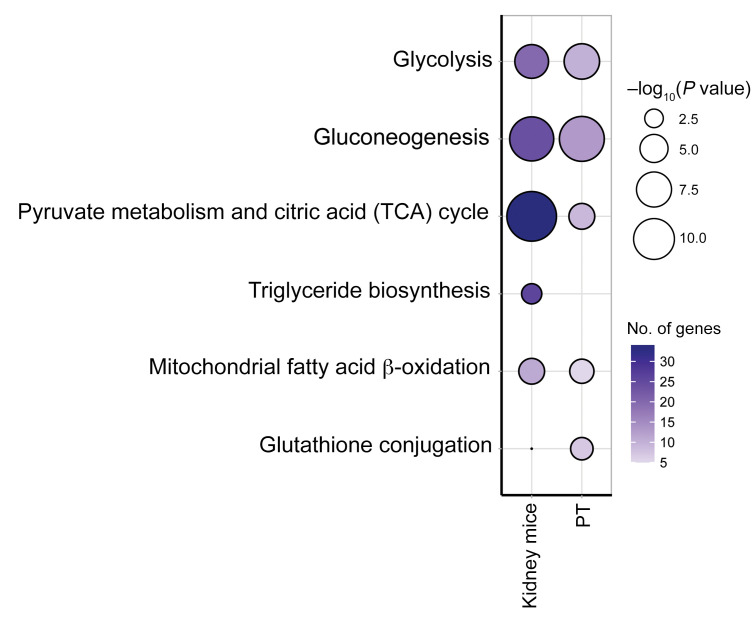
Transcriptomic alterations in mouse model of diabetes treated with SGLT2i validate alterations in central metabolic pathways in PT from humans. Dot plot comparing enriched pathways among suppressed transcripts in kidney cortex of SGLT2i-treated mice (*n* = 5 for each treatment group) with PT in T2Di(+).

Transcriptional data from kidney cortex of a murine model (17) were used to confirm the observed reversal of transcripts in metabolic pathways in PT cells with SGLT2i. Statistically significant transcriptional changes related to diabetes were validated by comparing uninephrectomized db/db mice with db/m mice. Reversals with SGLT2i were identified by comparing ReninAAV uninephrectomized db/db mice treated for 2 weeks with SGLT2i with untreated uninephrectomized db/db mice (17). Suppression of glycolysis, gluconeogenesis, TCA cycle, β-oxidation, and glutathione conjugation transcripts with SGLT2i observed in mouse cortex data was similar to that in human PT cells (Figure 6 and Supplemental Table 5). Given the preponderance of PT cells in the mouse cortex (>80%), the mouse transcriptional changes likely reflected changes in PT gene expression. Only metallothionein transcripts were enhanced in human PT data; however, no metallothionein transcripts were differentially expressed in the mouse data, precluding validation of this pathway. Supplemental Figure 8 shows transcript level alterations in human PT cells and mouse cortex with SGLT2i treatment.

### mTORC1 pathway as a mediator of effects of SGLT2 inhibition

**Figure 7:**
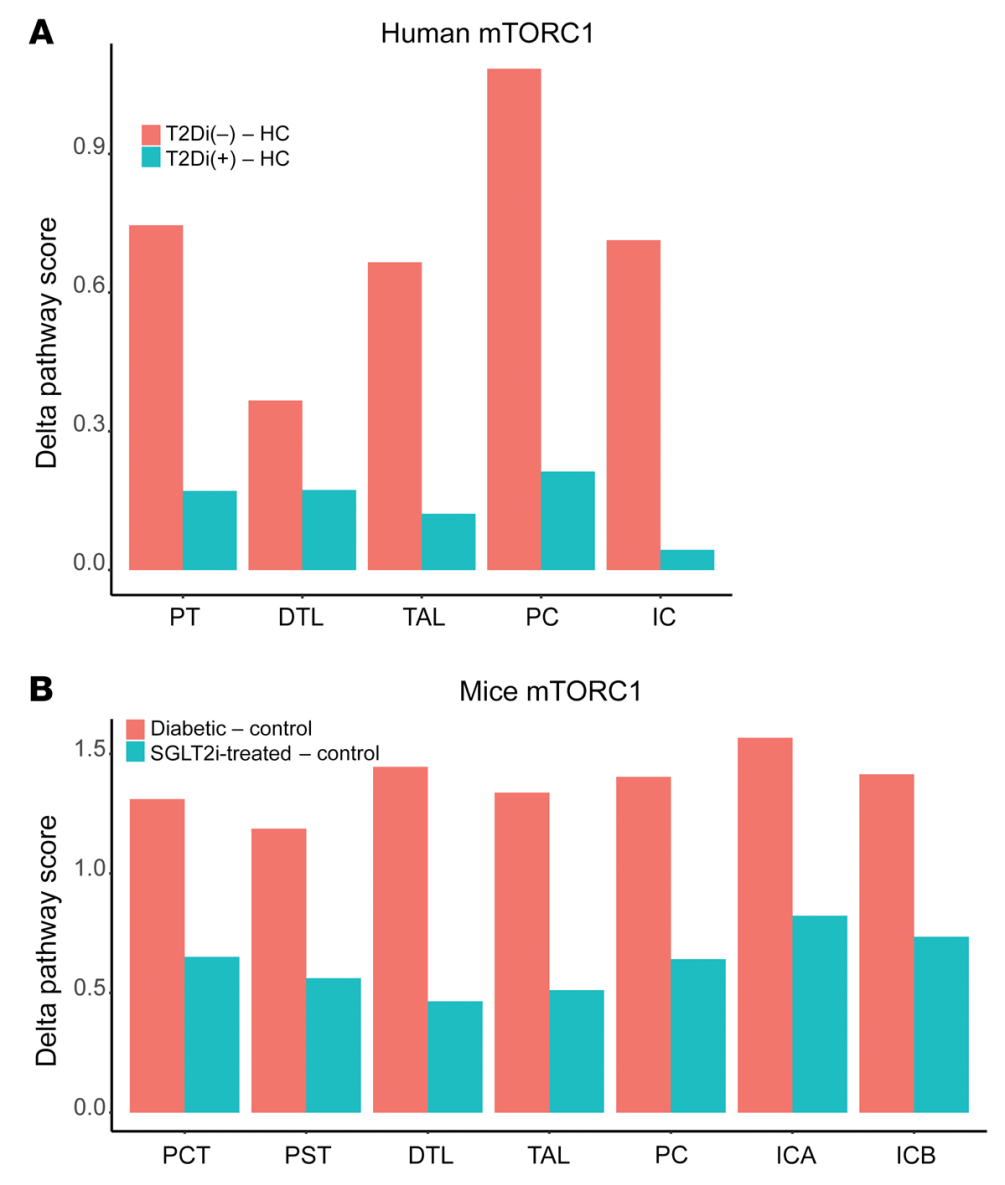
SGLT2i treatment–associated mTORC1 pathway score across tubular segments in human and mouse data. Using the Reactome database, 39 transcripts were associated with the mTORC1 pathway. (**A**) Plot represents the Δ mTORC1 pathway score by tubular segment as the difference between T2Di(–) and HCs (pink), and T2Di(+) and HCs (blue). (**B**) Similarly, in the mouse model snRNA-Seq data, Δ mTORC1 pathway scores were calculated in tubular segments as the difference between db/db mice (diabetic) and the background db/m (control) mice (pink) and SGLT2i-treated db/db/AAV and db/m (control) mice (blue).

SGLT2i suppressed transcripts related to metabolic pathways in all tubular segments except TAL. mTORC1 integrates cellular energy state signals, and therefore, diminished mTORC1 activity was hypothesized to be an intermediate regulator, mediating the effects of SGLT2i. The differences in mTORC1 pathway scores in T2Di(+) and T2Di(–) patients relative to HCs were computed and reported as the Δ mTORC1 pathway score (mTORC1 signaling cascade transcripts; Supplemental Table 6). SGLT2i treatment was associated with lower mTORC1 pathway scores in T2Di(+) compared with T2Di(–) patients in all tubular segments (Figure 7A). These findings were reproduced in the corresponding single-nucleus RNA-Seq (snRNA-Seq) data from the murine model (Figure 7B). Additionally, as mTORC1 is known to integrate signals from amino acid metabolism, transcripts associated with the amino acid metabolism pathway were among the significantly suppressed pathways with SGLT2i treatment in PT cells (Figure 4A).

**Figure 8:**
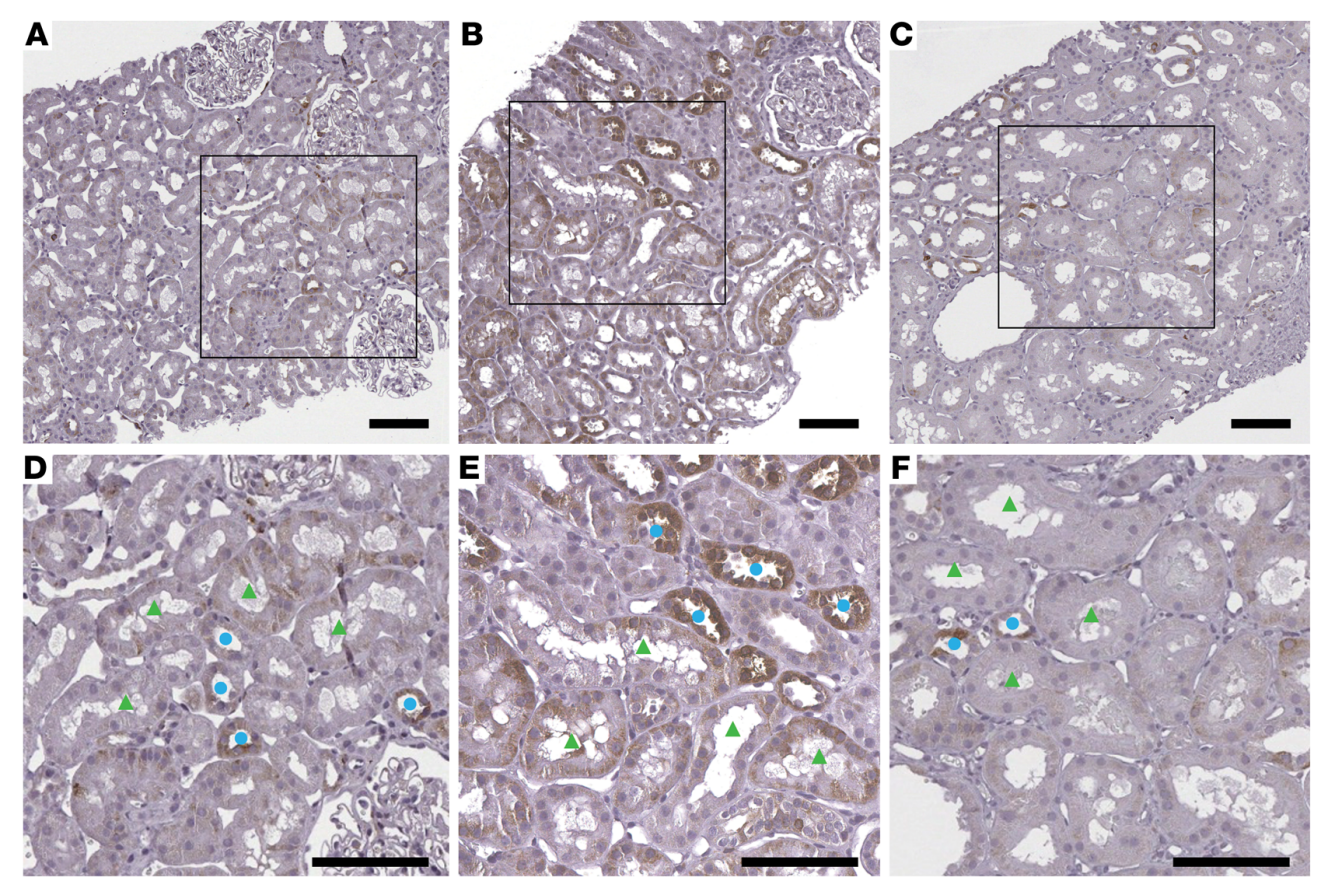
Phospho-S6 ribosomal protein immunohistochemistry. Representative images are shown from kidney biopsy sections immunohistochemically stained for phospho-S6 ribosomal protein from (**A**) HCs with average staining intensity for PT = 0.9 ( ± 0.4) and DT = 1.1( ± 0.5), (**B**) T2Di(–) with average staining intensity for PT = 1.4 ( ± 0.4) and DT = 2.1( ± 0.6), and (**C**) T2Di(+) with average staining intensity for PT = 0.8 ( ± 0.8) and DT = 1.1( ± 0.7), *n* = 4 in each group. Glass slides were scanned to whole slide images at ×40 magnification. Scale bars: 100 μm. Bottom panels **D**–**F** are closer views from square areas from panels **A**–**C**, respectively. Staining intensity was increased in both proximal (green triangles) and distal (blue circles) tubules in (**E**) T2Di(–) versus (**D**) HCs and was decreased in (**F**) T2Di(+). The average scores of the staining intensity were calculated from measurements by 2 independent pathologists.

As an independent measure of mTORC1 activity, levels of the downstream phosphorylated S6 (phospho-S6) protein were assessed by immunohistochemistry in kidney biopsy tissue sections (Figure 8). The average scores of the staining intensity from 4 study participants in each group by 2 independent pathologists for the proximal segments in HCs and T2Di(+) patients were similar at 0.9 and 0.8, while the T2Di(–) sections averaged 1.4 (Figure 8). The staining intensity scores for the distal segments were, on average, twice as high for T2Di(–) as T2Di(+) patients, which were closer to HCs, confirming reduction in mTORC1 activity with SGLT2i treatment in tubular segments of humans with T2D. Taken together, these results indicate that in the kidneys, treatment with SGLT2i in T2D decreases mTORC1 activity throughout the tubules, despite localized expression of SGLT2 in PT cells.

## Discussion

This study of young persons with T2D and early kidney injury demonstrates that treatment with SGLT2i was associated with significant transcriptional changes across virtually all tubular segments in the kidney, despite localization of SGLT2 expression to PT cells. Altered transcript levels found in T2D that were reversed with SGLT2i treatment were found in multiple metabolic pathways. The transcriptional alterations of metabolic pathways observed in PT in the human kidney were confirmed in kidney cortical tissue from an SGLT2i-treated DKD mouse model (17). This suggests that the transcriptional pathway changes identified in this cohort may also be relevant to alterations in later stages of DKD. In addition, mTORC1 signaling across all tubular cell types was significantly affected by SGLT2i. This finding was confirmed in the murine model and, at a protein level, in proximal and distal tubules in patients with phospho-S6 staining, which is consistent with changes in mTORC1-signaling activity. Taken together, these data are consistent with the hypothesis that SGLT2i mitigates perturbed cellular metabolic profiles and reduces mTORC1 signaling, a key driver of DKD, throughout the kidney tubules in early T2D (18–20).

SGLT2i treatment was also associated with lowering of glomerular volume and mesangial expansion compared with T2Di(–). Despite the small sample sizes for such comparisons, this observation is consistent with studies in rodent models (12). Conversely, no differences in fractional interstitial area were observed among the 3 groups, suggesting that transcriptomic differences observed in PT are not directly related to observable interstitial changes.

A rather interesting observation was that multiple metallothionein pathway transcripts in both proximal and distal nephron segments were consistently increased with SGLT2i. Metallothioneins are intracellular metal-binding proteins that mitigate damage from oxidative stress (21, 22). In streptozocin-diabetic mice, metallothionein deficiency has been shown to increase interstitial fibrosis and inflammatory interstitial infiltrates, which are important components of progressive DKD (23). Although mice also express metallothioneins, they were not enriched in the ReninAAV uninephrectomized db/db mouse model treated with SGLT2i. The metallothionein transcripts that were enhanced with SGLT2i in the human data have no known orthologs in mice, so our data may identify an additional pathway that mitigates oxidative stress in humans with SGLT2 inhibition. Murine studies suggest that SGLT2i mitigates oxidative stress via the glutathione pathway in the kidney cortex, particularly glomeruli, but not in the medulla (24). The single-cell resolution of our data suggests that glutathione conjugation pathway activity may differ between PT and TAL, which further supports cell type–specific differences in how the glutathione pathway attenuates oxidative stress with SGLT2i treatment.

There is increasing evidence that metabolic derangements and perturbed renal energetics are important drivers of DKD progression (25–28). The transcriptional changes observed between T2D patients and HCs align with recent evidence that diabetes increases glycolysis, β-oxidation, and TCA cycle activity in the kidney (26). One hypothesis posits that glomerular hyperfiltration in T2D exacerbates tubular workload by increasing the filtered Na^+^ load, thereby leading to increased ATP demand (3, 4, 29–35). The kidney relies heavily on β-oxidation to meet its high metabolic demands, although there are nephron segment–specific differences in metabolic processes and energy sources (25, 36, 37). Notably, PT has a greater capacity for gluconeogenesis than glycolysis compared with the distal nephron (38), which relies more on glycolysis and oxidative phosphorylation to meet its energy needs (39).

This study provides evidence that SGLT2 inhibition partly reverses the transcriptional expression of many metabolic pathway genes that are altered in youth-onset T2D with early kidney dysfunction. These findings suggest that some of the beneficial effects of SGLT2 inhibition on the kidney may occur by ameliorating some of the metabolic derangements present in T2D, as has been previously reported in animal studies (24, 26). Based on our data and mechanistic studies by others, we believe that transcriptional changes in the cells are related to changes in energy expenditure with SGLT2 inhibition. S1 and S2 proximal tubule cells have a high ATP demand for driving the basolateral sodium-potassium ATPase for maintaining solute transport. Glucose uptake and metabolism in PT cells derived from participants with diabetes treated with SGLT2i were distinct from that in those treated with other diabetic medications, despite similar glycemic control. For SGLT2i-naive individuals with diabetes, there are increased expression and activity of SGLT2 glucose transporters in PT cells to allow for increased glucose uptake on the apical lumen (40). Due to the mitochondrial dysfunction in kidneys associated with diabetes, the increased transport via glucose transporters may drive the need for enhanced glycolytic flux seen in PT cells to maintain transport capabilities (26). In contrast, with SGLT2i, there would be substantial reduction in uptake and transepithelial transport of glucose compared with that in T2Di(–) patients or HCs. The resultant decrease in glucose uptake in PT likely explains the myriad metabolic effects, including the observed reduction in expression of glycolytic and TCA cycle transcripts. Since the large numbers of high capacitance, facilitative glucose transporters on the basolateral membrane of PT cells tends to “clamp” intracellular glucose concentrations at or slightly above systemic glucose levels (41, 42), the transcriptional changes observed in our study potentially correspond primarily to changes in glucose uptake and reabsorption rather than intracellular glucose levels.

This study highlights the nephron segment specificity in metabolism-related gene expression that occurs even in anatomically contiguous segments. For example, SGLT2i treatment enhanced expression of genes involved in glycolysis, TCA cycle, and β-oxidation in TAL. The results from this study suggest that TAL might have different energy requirements with SGLT2i. TAL primarily takes in glucose via GLUT1 on the basolateral membrane (41). Therefore, changes in extracellular glucose levels have essentially no impact on glucose uptake in TAL, since GLUT1 transporters are high affinity transporters that are already at or near maximal activity at physiological glucose levels (41). Moreover, TAL has increased capacity for glycolysis compared with PT cells (38). As TAL is the next nephron segment with sodium-resorbing capabilities downstream of PT, the transcriptional differences in this study suggest that TAL might have increased energy requirements with SGLT2 inhibition due to the increased sodium load in the distal tubular lumen, consistent with previous mathematical models (32, 43). Further mechanistic studies will be needed to determine whether the observed changes in distal nephron segments contribute to the overall nephroprotective effect seen with SGLT2i.

An advantage of transcriptional profiles is the opportunity to identify key upstream regulators of the gene expression responses to an external stimulus, using the mRNA signatures as a pathway readout. Such an approach identified mTORC1 as a likely driver of gene expression changes. mTORC1, a protein kinase, is a key signaling hub that integrates signals from nutrient metabolism pathways, particularly those of glucose and branched-chain amino acids, and in turn regulates lipid metabolism, cell proliferation, cell growth, and autophagy with a coordinated transcriptional response (44). Increased mTORC1 activity plays an important pathogenic role in podocyte dysfunction in DKD (20), is implicated as a contributor to kidney hypertrophy along the nephron, and is known to induce a proinflammatory phenotype (45). In a recent study using the Akita mouse model of diabetes, SGLT2i decreased mTORC1 signaling in proximal tubular cells (19). Furthermore, increased mTORC1 activity was sufficient to abolish the beneficial effects of SGLT2i, thereby suggesting that mTORC1 signaling may mediate many of the protective effects of SGLT2i by preventing the development of fibrosis (19). Using the differences in mTORC1 transcriptional scores between the T2D and HC groups in human and mouse data, accompanied by phospho-S6 staining across tubular segments, we provide evidence for reduced mTORC1 activation in proximal and distal tubular segments in youth with T2D treated with SGLT2i. Further mechanistic work is needed to explain the observed decrease in mTORC1 activity in both the proximal and distal nephron segments given that PT presumably has decreased energy expenditure and TAL has increased energy demand with SGLT2i.

SGLT2i clearly helps prevent kidney disease progression in older individuals with established DKD (9, 11, 46, 47). However, the participants in the current study had youth-onset T2D, an emerging epidemic with a high risk of early-onset DKD in whom the effects of SGLT2i are not established (48). Indeed, based on data about the incidence of microvascular complications from the TODAY2 study, there is a high likelihood that the majority of these participants will develop signs of DKD, including albuminuria (48, 49). At the time of kidney biopsy, some of these participants already exhibited early signs of kidney dysfunction, including elevated eGFR and albuminuria. While our sample size was too small for formal analyses, morphometric data also suggested early structural changes, including increased glomerular volume and mesangial expansion, which are associated with future loss of kidney function (50). These data provide important insights into the transcriptional alterations associated with SGLT2i in a high-risk population early in the disease process. The effects typically associated with longstanding disease and associated comorbidities on structural and functional changes such as interstitial fibrosis in kidneys are minimal in these young participants. Whether these transcriptional changes also affect structural and functional features of longstanding disease is unknown. The data from the murine model of advanced DKD suggest that they may.

To our knowledge, this is the first study that interrogated research kidney biopsies in youth-onset T2D to examine differences in intrarenal, cell type–specific transcriptomic signatures associated with SGLT2i treatment. The single-cell resolution of these data provided unique insights into cell type–specific responses and enabled the assessment of nephron segment–specific responses in a population of youth with T2D who are at high lifetime risk of developing DKD.

The study has several constraints, including the cross-sectional and observational examination, which limit causal inferences of SGLT2i treatment, and the relatively small sample size. For this reason, these data presented should be considered hypothesis generating. Due to the observational nature of the cohort study, residual confounding that may have biased treatment groups cannot be excluded. While we do not currently have long-term outcomes regarding the kidney health of these participants, such data are being collected. To address these limitations, we were able to validate the differentially expressed pathways and directionality of their changes using publicly available murine data. The murine model of DKD was more advanced than the human samples, and different RNA-Seq technologies were used, limiting the conclusions to be drawn from this comparison. Further studies are needed to compare human data with data from murine models of SGLT2i to further characterize shared and specific changes between mice and humans. Additionally, our results show small absolute transcriptional changes at multiple steps in metabolic pathways and do not include metabolic flux data for these pathways. However, we have previously shown that relatively modest transcriptional changes in metabolic pathways correspond to biologically meaningful changes in metabolic flux in DKD (26) and that transcript abundance in scRNA-Seq data correlates with protein and metabolite levels (51). Additionally, the change in staining intensity of phospho-S6 supports the concept that such changes result in biologically meaningful changes at a functional level. Finally, scRNA-Seq data may not capture genes that are expressed at a low level and in rare cell types, which may hinder our ability to classify cells, including rare cell types, such as juxtaglomerular cells and type B ICs. Although transcriptional changes may reflect the cell type–specific response to changes in metabolites (26), further investigations with metabolome measurements in this cohort would help to determine the ultimate impact of SGLT2i on the metabolic flux in the kidney. Spatial metabolomic techniques (52) could also provide more insights on the nephron segment–specific changes, particularly when integrated with other novel tissue-interrogation technologies (53).

In conclusion, young persons with T2D and early kidney injury exhibited transcriptional signatures of perturbed renal metabolism, which were attenuated in the presence of SGLT2i. Most transcriptional changes associated with SGLT2i were in the distal tubular segments where *SLC5A2* is not expressed. Although further investigations are needed to identify the mechanisms responsible for the changes in the distal nephron segments, our data suggest that alterations in the mTORC1 pathway may mediate some of these transcriptional changes. Future directions include designing a rigorous trial to examine the molecular and metabolic mechanisms by which SGLT2 inhibition mitigates the progression of DKD in T2D as well as determining whether the transcriptional changes observed in this study predict long-term progression of kidney injury and functional decline.

## Methods

### Study design and participants

Adolescents and young adults (*n* = 16) with T2D (12–21 years of age, T2D onset <18 years of age,T2D duration 1 to 10 years, and HbA1c <11%) from the Renal HEIR (NCT03584217) and the IMPROVE-T2D (NCT03620773) studies who volunteered for a nested protocol research kidney biopsy were included in this analysis (Figure 1). The participants were recruited from the Type 2 Diabetes and Metabolic Bariatric Surgery clinics at the Children’s Hospital Colorado at the Anschutz Medical Campus in Aurora, Colorado, USA. T2D was defined by American Diabetes Association criteria plus the absence of glutamic acid decarboxylase, islet cell, zinc transporter 8, and/or insulin autoantibodies. Exclusion criteria are detailed in Supplemental Table 1. The Renal HEIR and IMPROVE-T2D cohorts have intentionally harmonized study protocols. Medication use was recorded for all participants, and T2D treatment, including SGLT2 inhibition, was at the discretion of their medical provider. Normative reference tissue research biopsies were provided by 6 healthy young adult participants in the CROCODILE study (NCT04074668).

### Clinical measurements

All laboratory assays for the Renal HEIR, IMPROVE-T2D, and CROCODILE cohorts were performed by the University of Colorado Clinical and Translational Research Centers (CTRC) Core Labs and the NIDDK laboratory in Phoenix, Arizona, USA. Other fasting laboratory evaluations included the following: total cholesterol, LDL cholesterol (LDL-C), HDL cholesterol (HDL-C), triglycerides, glucose, and HbA1c (DCCT-calibrated). Assays were performed by standard methods in the CTRC laboratory.

#### Ultrasound-guided kidney biopsies and tissue processing.

All 3 studies (i.e., Renal HEIR, IMPROVE-T2D, and CROCODILE) use the same pathology protocol as KPMP. Briefly, an ultrasound-guided percutaneous kidney biopsy was performed by 1 of 2 highly experienced interventional radiologists. Per local protocol, up to 4 passages were performed to obtain 3 biopsy cores. Each core was immediately assessed for the presence of cortex by gross examination and digital imaging. Kidney tissue was placed in specific fixatives and shipped to the University of Michigan (54).

### Quantitative morphometrics

Light microscopy sections were assessed for pathologic diagnosis. For quantitative assessment of glomerular and mesangial volume and mesangial nuclear count, all glomeruli present in a 3 μm formalin-fixed paraffin-embedded section, stained with periodic acid–Schiff, of each specimen were assessed using quantitative morphometrics as previously described (55, 56). Mesangial index was percentage of mesangial area per glomerular volume.

### Sample processing and scRNA sequencing

scRNA-Seq profiles were obtained using KPMP protocols (54). Briefly, single cells were isolated from frozen tissues using Liberase TL at 37°C for 12 minutes. The single-cell suspension was immediately transferred to the University of Michigan Advanced Genomics Core facility for further processing. Sample demultiplexing, barcode processing, and gene expression quantifications were performed with the 10x Genomics Cell Ranger, version 6, pipeline using the hg38 GRCh38-2020-A reference genome (57–59). To remove ambient mRNA from the data, the Cell Ranger count matrices were processed using SoupX (60) with the default parameters. The resulting matrices were processed as previously described (61), with cells included only if gene counts were between 500 and 5,000, with fewer than 50% mitochondrial genes. Individual matrices were then integrated using RunHarmony embedded in Seurat, version 4.0.0. Clusters were annotated based on previously established kidney cell markers (13, 61) (Supplemental Figure 2A). Data were deposited in the NCBI’s Gene Expression Omnibus database (GEO GSE220939).

#### Murine models.

To validate the findings from the human data, published bulk (GEO GSE199437) and snRNA-Seq (GEO GSE184652) data from the kidney of mouse models (17) of diabetes were used. In brief, female db/db mice (BKS.Cg-Dock7m +/+ Leprdb/J from The Jackson Laboratory, BKS background, lot 642) underwent left nephrectomy at 5 weeks of age and at 12 weeks were infected with renin-expressing adeno-associated virus (ReninAAV) (109 genomic copies), which accelerates disease progression. ReninAAV uninephrectomy db/db mice treated with SGLT2i for 2 weeks (SGLT2i-treated diabetic mice) were compared with untreated uninephrectomy db/db mice (diabetic mice) and db/m (control) mice.

### Immunohistochemistry for phospho-S6 ribosomal protein

Sections were dewaxed, rehydrated, and subjected to heat-induced antigen retrieval by incubating in 0.1 M citrate buffer (pH 6.0, Abcam, catalog ab93678). Endogenous peroxidase was blocked with 3% H_2_O_2_ (MilliporeSigma, catalog H1009) in TBS (pH 7.4) for 30 minutes. Sections were then blocked with 10% normal goat serum in 5% BSA at room temperature for 2 hours. Phospho-S6 ribosomal protein (Ser240/244) rabbit mAb (Cell Signaling Technology, catalog 5364) was used at a concentration of 1:1,000 in a cold room (4°C) overnight. Antibody binding was detected by using secondary antibody, goat anti-rabbit Ig-HRP (1:100, Southern Biotech, catalog 1010-05), and the Betazoid DAB Chromogen Kit (Biocare Medical, catalog BDB2004). Counterstaining was performed using hematoxylin. Two pathologists who were blinded to the participants’ clinical data scored the intensity of the phospho-S6 staining as follows: 0 indicated less than 5% of cells stained, 1 indicated from 5% to 25% of cells stained, 2 indicated from 25% to 50% of cells stained, and 3 indicated more than 50% of cells stained. Separate scores were created for the proximal and distal nephron segments, and the scores of the 2 pathologists were averaged.

### Statistics

#### Groups.

Participants were stratified as follows: T2D without SGLT2i (T2Di[–]), T2D with SGLT2i (T2Di[+]), and normative reference tissue from HCs. Additionally, the term T2D indicates all participants with T2D irrespective of SGLT2i use. Statistical power was limited to compare baseline clinical and morphometric comparisons between biopsy groups; thus, quantitative data are presented as mean and SD.

#### Differential expression analysis.

To identify the transcripts potentially influenced by SGLT2 inhibition, the Limma R package (62) was used to fit linear regression models with the Benjamini-Hochberg (BH) procedure to correct for multiple testing. The fold changes (log_2_FC) were calculated between 2 comparisons: T2Di(–) versus HC and T2Di(+) versusT2Di(–). Transcripts were required to pass FDR-adjusted *P* values of less than 0.05 in T2Di(–) versus HC and in T2Di(+) versus T2Di(–) to be considered “reversed”. A T2D reversed transcript was considered to be as follows: (a) “suppressed” with SGLT2i if the log_2_FC of the transcript level in T2D relative to HC was greater than 0 and log_2_FC T2Di(+) relative to T2Di(–) was less than 0 and (b) “enhanced” with SGLT2i if its log_2_FC in T2Di(–) relative to HC was less than 0 and log_2_FC T2Di(+) relative to T2Di(–) was greater than 0 (Supplemental Figure 4A).

#### Enrichment analysis.

Enrichment for transcripts reversed by the SGLT2i was determined using the enrichR package (63–65) and the Reactome database (66–68). A pathway was considered significant if its *P* value was less than 0.05 and it included at least 5 transcripts reversed by SGLT2i. Based on existing literature, genes and their Reactome terms were further categorized manually into smaller groupings.

#### Protein-protein interaction network.

The StringApp, version 1.7.1, implemented in Cytoscape, version 3.7.2 (69), was used to generate the protein-protein interaction networks for reversed genes.

#### MTORC1 pathway score.

The single-sample gene set enrichment analysis (ssGSEA) method implemented in the GSVA R (70) package, version 1.40.1, was used to compute the mTORC1 pathway score using the transcripts in this pathway listed in the Reactome database (R-HSA-165159, Supplemental Table 6).

#### Analysis of mouse data.

Publicly available data (GEO GSE199437) from mouse cortex (17) were analyzed using the identical reversal analysis approach (Supplemental Figure 4A) and definitions described for human gene expression studies to identify transcripts suppressed/enhanced with SGLT2i to validate the findings in the human PT cells. The analogous treatment groups were as follows: db/m mice (group 1 in the GEO data set) for controls; db/db + uninephrectomy + PBS (group 2) for diabetic mice; and db/db + uninephrectomy + reninAAV + SGLT2i for 2 weeks (group 7B) for SGLT2i-treated diabetic mice. Limma R package (62) for DEG identification, BH for multiple testing correction, and an adjusted *P* value of 0.05 or less were similarly applied. Effects of SGLT2i on metabolic pathways were assessed in the murine data after applying the same analytical approach and criteria for defining reversed, suppressed, and enhanced genes as used in the human data. The mTORC1 pathway score was assessed using publicly available single nucleus data (GEO GSE184652) from the same murine models as the bulk RNA-Seq analysis (17). The same analogous treatment groups as used for the bulk data and analytical approach described for the human data were applied to snRNA-Seq murine data to determine the Δ mTORC1 pathway score.

### Study approval

All 3 studies, CROCODILE, Renal HEIR, and IMPROVE-T2D, were approved by the Colorado Multiple Institutional Review Board (COMIRB). Written, informed consent was received from each participant and/or parents as appropriate for age prior to enrollment. Participants who opted to undergo the optional kidney biopsy were specifically and additionally consented by the research and biopsy teams.

## Author contributions

The 4 first coauthors each made unique and critical contributions to this manuscript, and authorship order was determined after discussion among writing group members. FMA, KI, JBH, SE, PJM, PB, ASN, JAS, MK, and RGN designed the study. PB, MK, and RGN acquired funding for the study. PB led the cohort studies. CLO, MB, DD, JBH, PB, EAO, RH, and PL acquired or generated the data. JAS, ASN, FMA, SE, PJM, LP, PB, RM, JH, DF, and VN analyzed the data. JAS, FMA, PJM, ASN, LS, LP, PB, MK, SE, FCB, and RGN wrote the manuscript. SP, KI, JBH, FCB, RGN, MK, and PB provided scientific guidance and insights. All authors reviewed, edited, and approved the manuscript.

## Supplementary Material

Supplemental data

Supplemental table 2

Supplemental table 3

Supplemental table 4

Supplemental table 5

## Figures and Tables

**Table 2:**
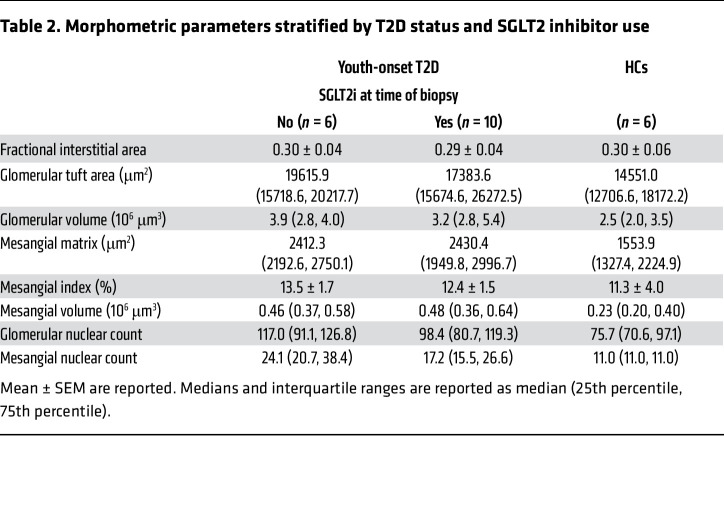
Morphometric parameters stratified by T2D status and SGLT2 inhibitor use

**Table 1:**
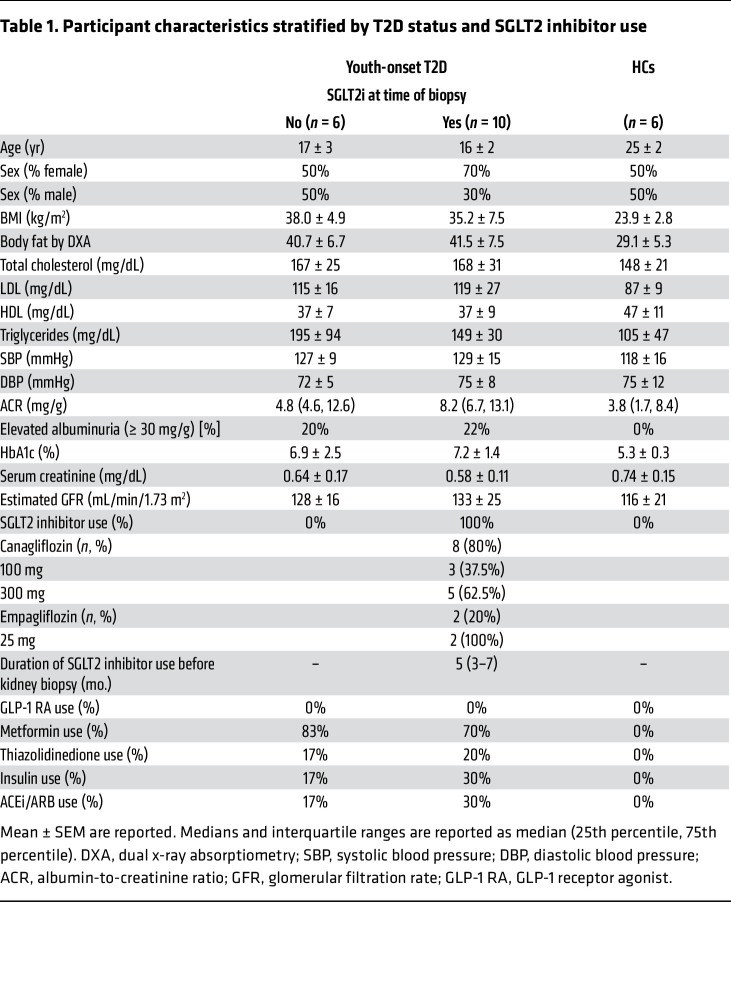
Participant characteristics stratified by T2D status and SGLT2 inhibitor use

## Version 1 01/13/2023

In-Press Preview

## Version 2 03/01/2023

Electronic publication
