# Supplementary material for: SGLT2 inhibitors mitigate kidney tubular metabolic and mTORC1 perturbations in youth-onset type 2 diabetes
Source: J Clin Invest. 2023 Mar 1;133(5):e164486. doi: 10.1172/JCI164486 (PMC9974101; doi:10.1172/JCI164486)
Supplement: Supplemental data [file jci-133-164486-s079.pdf]

Supplementary Figure S1. Images of representative glomeruli of A) healthy controls, B) T2D without SGLT2i treatment [T2Di(-)], and C) T2D with SGLT2i treatment [T2Di(+)] from PAS-stained 2-3 micrometer thick formalin-fixed, paraffin-embedded sections. Scale bars are 50 micrometers.

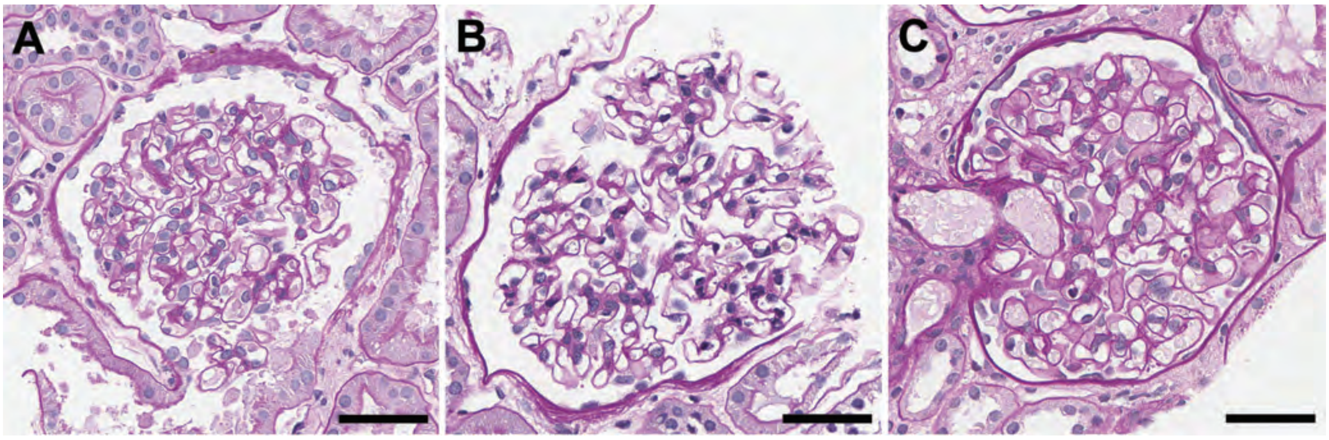

Supplementary Figure S2: Quality control for single-cell RNA sequencing

A) Cell specific markers used to define cell clusters. B) HC, T2Di(-) and T2Di(+) contributed to each cell cluster. Abbreviations: PT - proximal tubule, DTL - descending thin limb, ATL - ascending thin limb, TAL- thick ascending limb, DCT - distal convoluted tubule, CNT - connecting tubule, IC - intercalated cells, PC - principal cells, tPC-IC transitioning intercalated/principal cells, EC - endothelial cells, vSMC/MC/Fib - vascular smooth muscle cells/mesangial cells/fibroblasts, PEC - parietal epithelial cells, POD - podocytes, MAC - macrophages, MON - monocytes, B - B cells, NKT/NKCT - Natural Killer T Cells/Natural Killer Cells with T cells

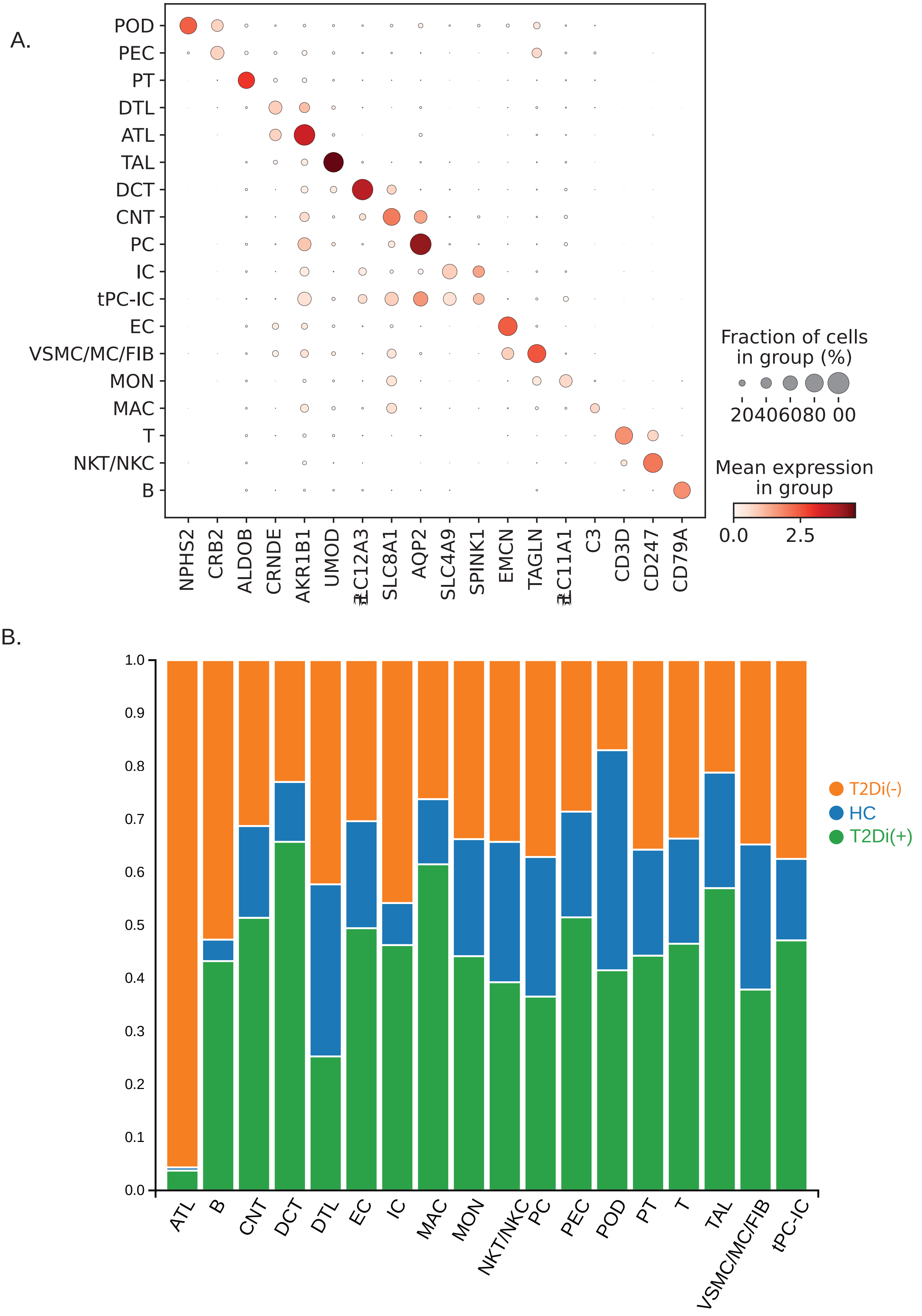

Supplemental Figure S3. Anatomic and cell state annotations for proximal tubule sub-clusters

A-C) Transcriptional profiles from cells from HC, T2Di(-), or T2Di(+) were analyzed for proximal tubule (PT) biomarkers for compartments S1, S2, or S3, from the KPMP Consortium*. Compartment-specific transcripts were more abundant in clusters PT-1 through PT-3 (**A**) than PT-4 (**B**) or PT-5 (**C**). **D**) Adaptive/maladaptive state biomarkers defined by KPMP for PT-1 through PT-5. **E**) Cell cycling state biomarkers defined by KPMP for clusters PT-1 to PT-5. **F**) Degenerative state biomarkers from the KPMP for clusters PT-1 through PT-5.

*-Lake BB et al. bioRxiv. 2021:2021.07.28.454201.

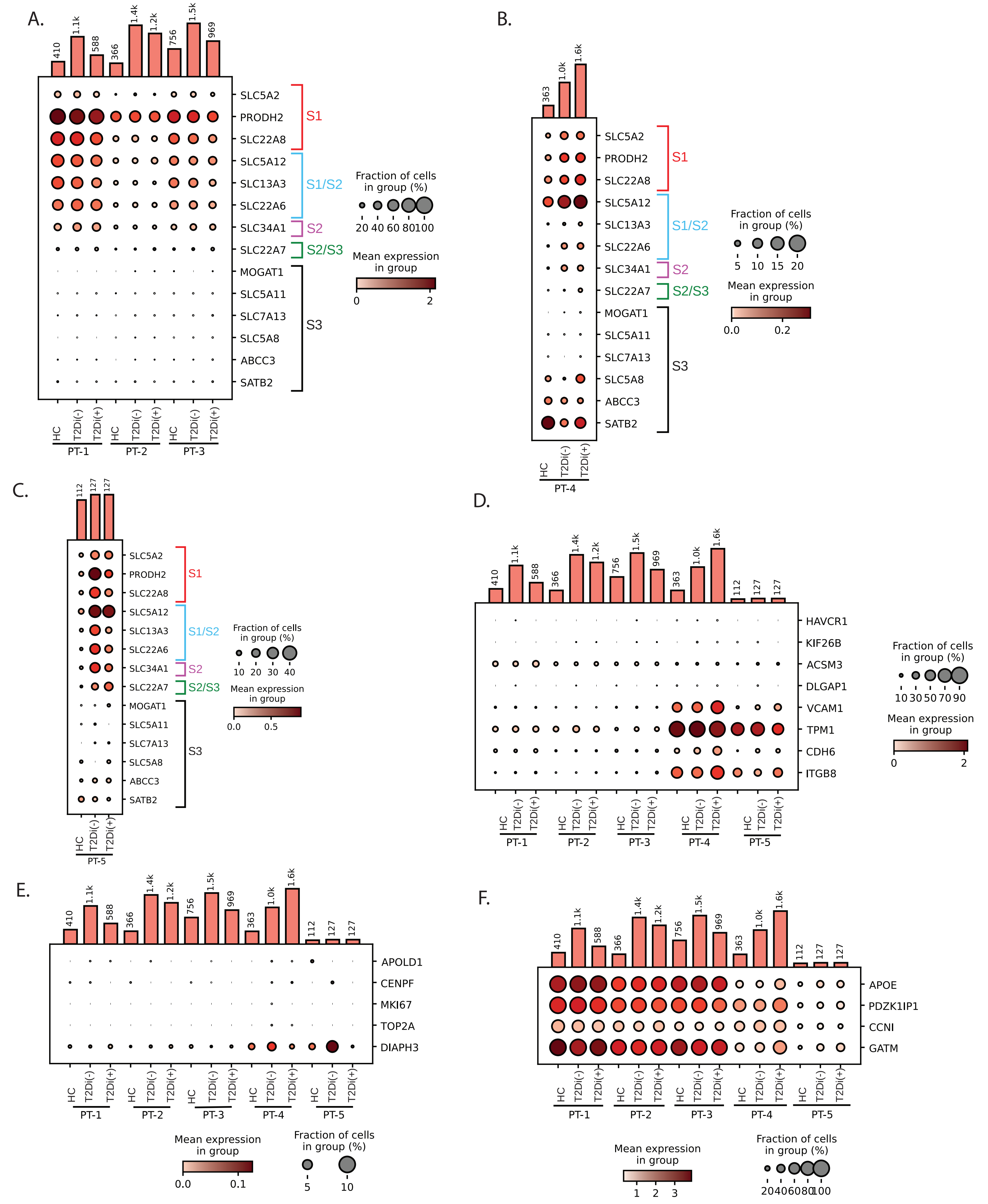

Supplementary Figure S4. A) Bioinformatic pipeline describing the analysis of scRNAseq data from the three groups, HC, T2Di(-) and T2Di(+). **B)** Disease associated transcriptional differences when comparing T2Di(-) and HC.

A. Bioinformatic pipeline

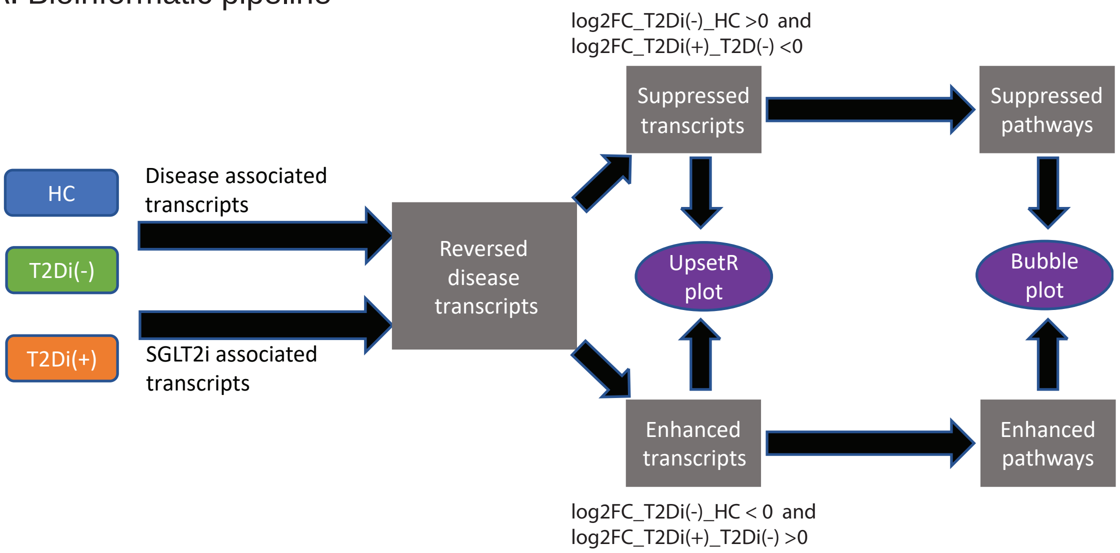

B. Altered expression in type 2 diabetes (T2Di(-) vs HC)

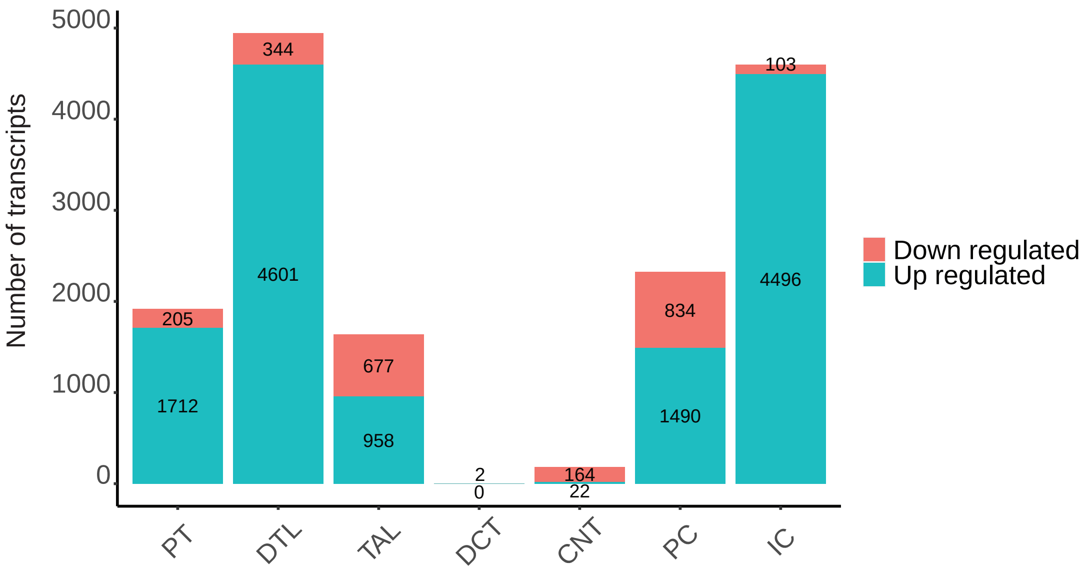

Supplementary Figure S5: Protein-protein interaction networks of transcriptional alterations in PT with SGLT2 inhibition. Protein-protein interaction networks in all PT clusters comparing A)T2Di(-) to HC, B)T2Di(+) to T2Di(-) and C) T2Di(+) to HC. Each dot represents an individual gene and is color coded for any gene with a significant logFC (red: 0 to 0.1 and blue: -0.1 to 0 logFC). Comparison between T2Di(+) to HC (panel C) shows that SGLT2 inhibition retains some differences from HC.

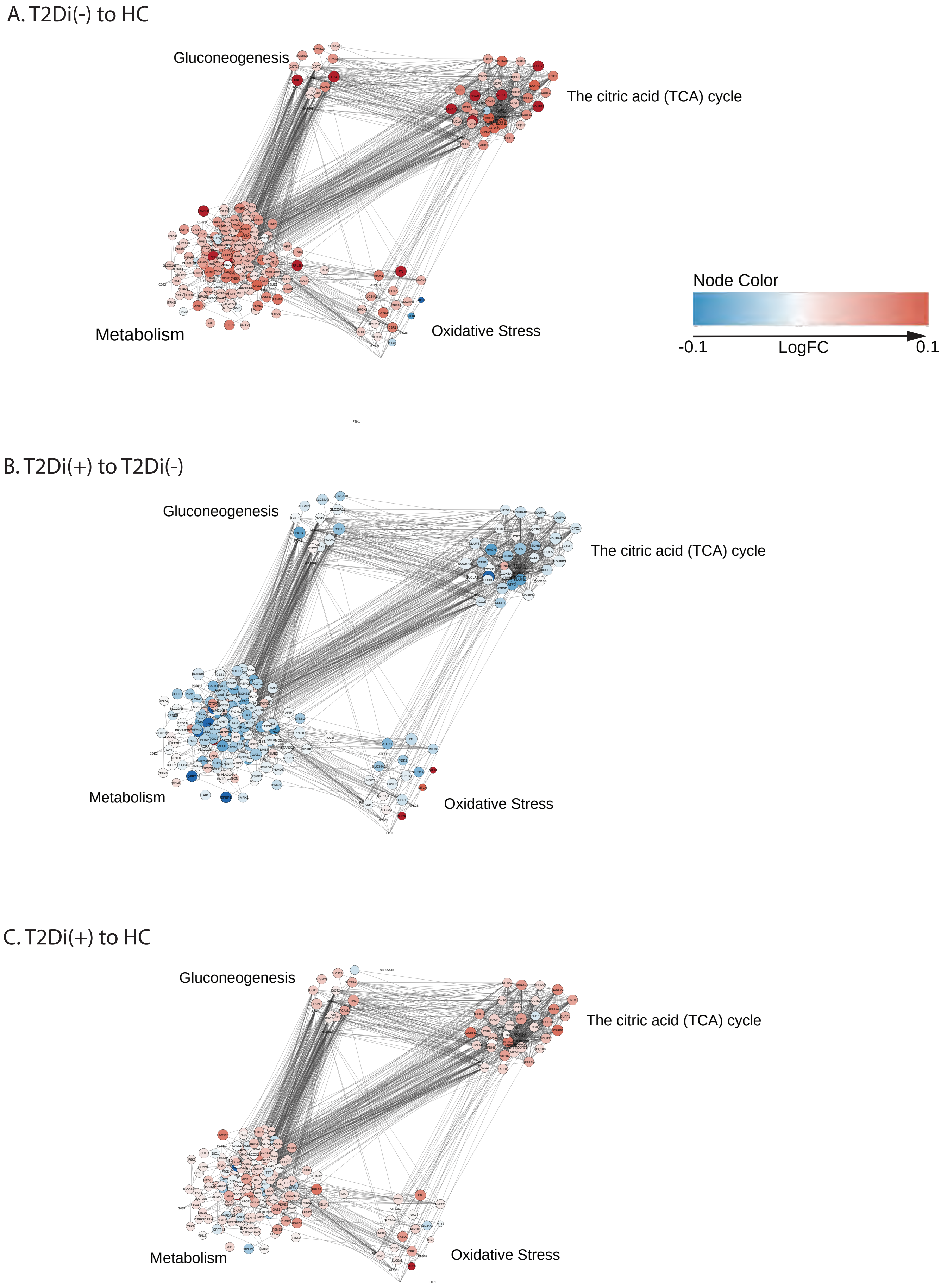

Supplementary Figure S6: Differential effects with SGLT2i on PT cells, with and without SLC5A2 expression. A) Enriched pathways from transcripts suppressed with SGLT2i in PT cells expressing SLC5A2 expression (PT+). B) Enriched pathways from transcripts suppressed with SGLT2i in PT cells with no detectable SLC5A2 expression (PT-). C) Enriched pathways from transcripts enhanced with SGLT2i in PT- cells. There were too few enhanced transcripts with SGLT2i in PT+ cells for an enrichment analysis.

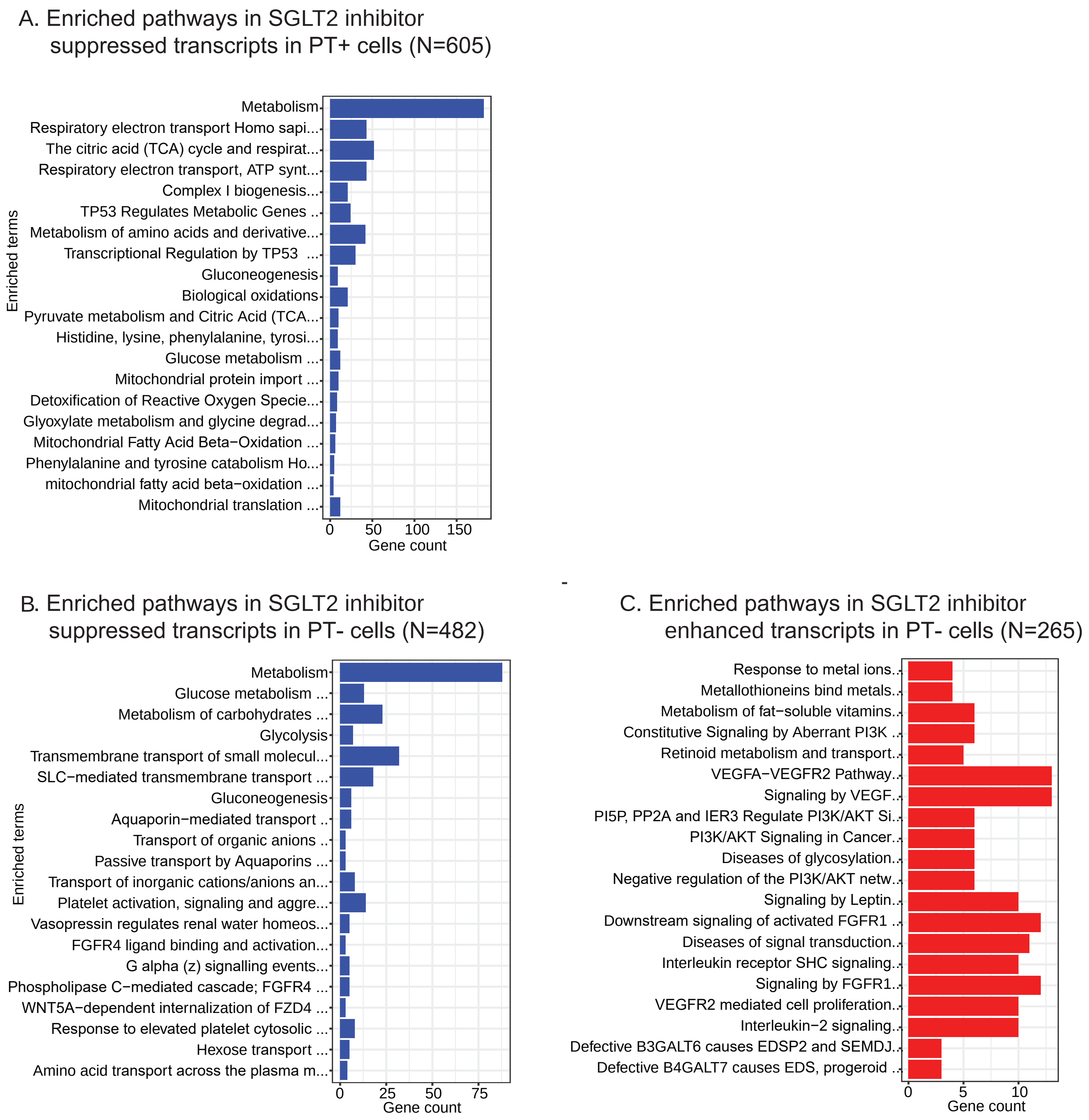

Supplementary Figure S7: Reactome enrichment analysis for: A) enhanced transcripts in DTL; B) suppressed transcripts in DTL; C) enhanced transcripts in PC; D) suppressed transcripts in PC; E) enhanced transcripts in IC; and F) suppressed transcripts in IC

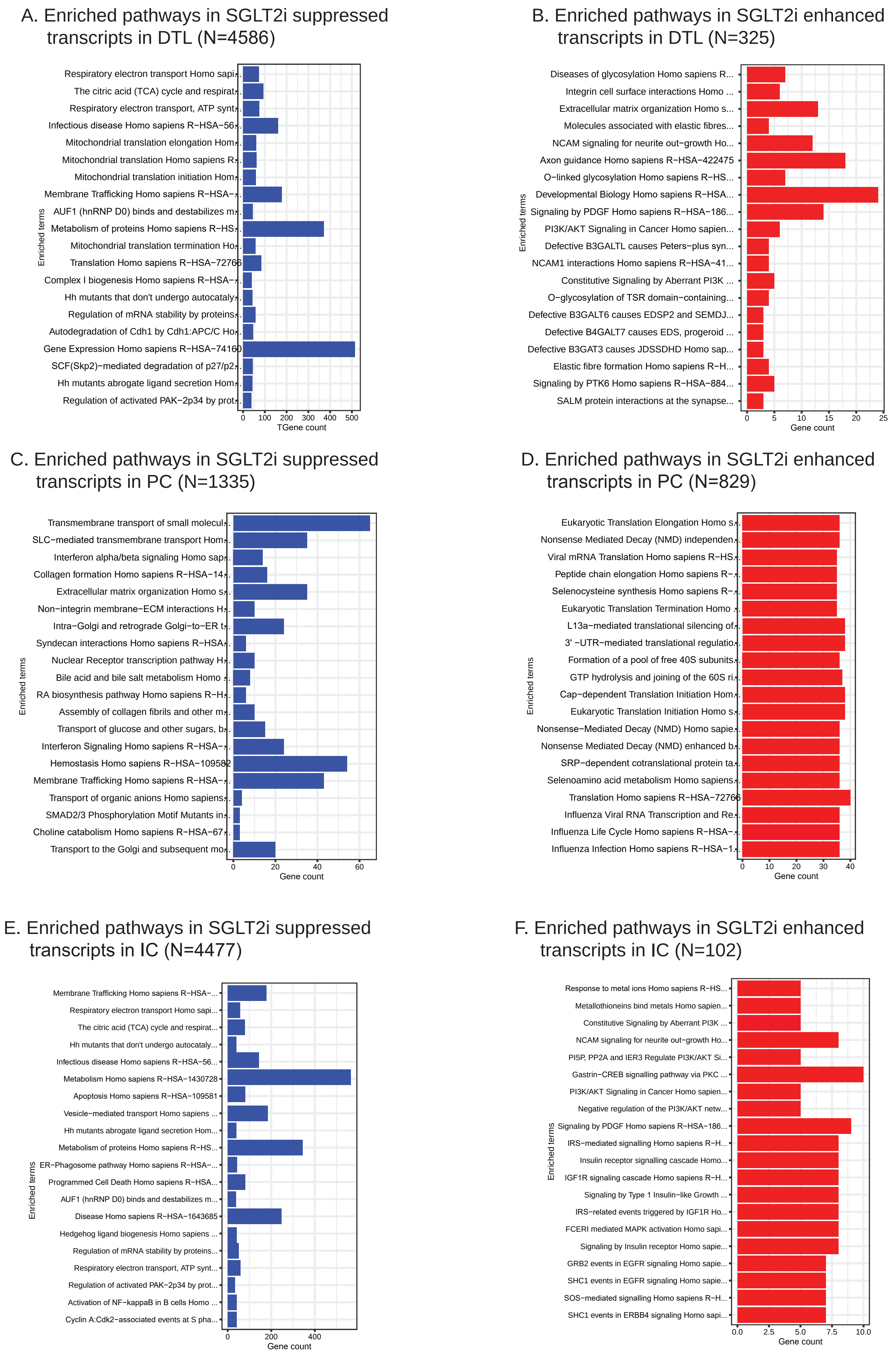

Supplementary Figure S8: Transcriptional alterations in human PT cells and mouse kidney cortex, with SGLT2 inhibitors. Significantly perturbed transcripts in central metabolism pathways were similar in human PT cells and mouse kidney cortex with SGLT2i treatment. In mice, db/m=non-hypertensive, non-diabetic control mice; db/db= diabetic mice; SGLT2i=SGLT2i treated diabetic mice.

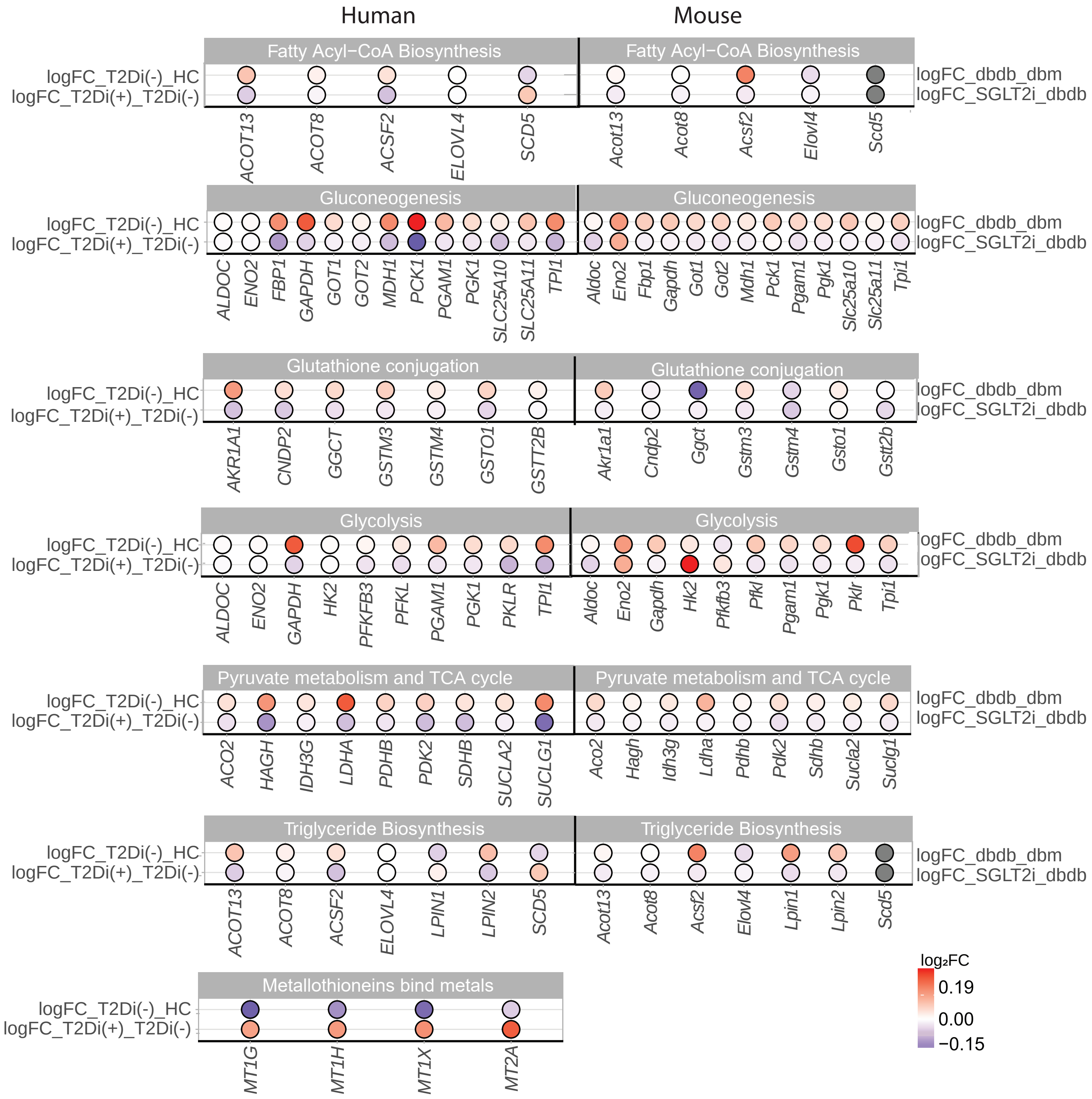

Supplementary Table S1. Exclusion criteria for percutaneous kidney biopsy

Additional exclusion criteria for those undergoing kidney biopsy
Evidence of bleeding disorder or complications from bleeding
Use of aspirin, NSAIDS or other blood thinner that cannot be safely stopped for a sufficient time period before and after the biopsy so as to add no additional risk of bleeding
Blood urea nitrogen (BUN) > 80 gm/dL
INR > 1.4
PTT > 35 seconds
Hemoglobin (Hb) < 10 mg/dL
Platelet count < 100,000 / µL
Uncontrolled or difficult to control hypertension (> 150/90 mmHg at the day of biopsy)
eGFR < 40 mL/min/1.73m^2
Single kidney (either by history, documented by prior imaging or ultrasound performed prior to the biopsy)
> 2 cm discrepancy between left and right kidney sizes based on largest longitudinal diameter determined by US performed prior to the biopsy.
Kidney size: One or both kidneys < 9 cm
Hydronephrosis or other important renal ultrasound findings such as significant stone disease
Any evidence of a current urinary tract infection as indicated on day of biopsy
Clinical evidence of non-diabetic renal disease
Positive urine pregnancy test or pregnancy

Supplementary Table S6: List of MTORC1 pathway List of MTORC1 pathway transcripts, based on Reactome database (R-HSA-165159), used in determining pathway score.

mTOR signalling Homo sapiens R-HSA-165159 from REACTOME
STRADA
STRADB
RPS6
TSC2
TSC1
PRKAB1
PRKAB2
MTOR
PPM1A
RRAGB
RRAGA
RRAGD
RHEB
RPS6KB1
RRAGC
AKT1S1
EIF4G1
LAMTOR1
LAMTOR3
LAMTOR2
LAMTOR5
LAMTOR4
EIF4B
PRKAA2
YWHAB
CAB39L
CAB39
EIF4EBP1
PRKAG2
PRKAG3
PRKAG1
PRKAA1
STK11
RPTOR
EEF2K
MLST8
AKT1
AKT2
EIF4E
